# Magnetic Iron Oxide Nanoparticle (IONP) Synthesis to Applications: Present and Future

**DOI:** 10.3390/ma13204644

**Published:** 2020-10-18

**Authors:** Nene Ajinkya, Xuefeng Yu, Poonam Kaithal, Hongrong Luo, Prakash Somani, Seeram Ramakrishna

**Affiliations:** 1 Materials and Interfaces Center, Shenzhen Institutes of Advanced Technology, Chinese Academy of Sciences, Shenzhen 518055, China; xf.yu@siat.ac.cn (X.Y.); hluo@scu.edu.cn (H.L.); 2 Department of Molecular and Cellular Engineering, Jacob Institute of Biotechnology and Bioengineering, SHUATS, Allahabad 211007, India; poonam.kaithal@gmail.com; 3 Center for Grand Challenges and Green Technologies, Applied Science Innovations Pvt. Ltd., Pune 411041, India; spsomani97@gmail.com; 4 Center for Nanofibers and Nanotechnology, National University of Singapore, Singapore 117576, Singapore; seeram@nus.edu.sg

**Keywords:** iron oxide nanoparticles (IONPs), formation mechanisms, reproducible, biomedical

## Abstract

Iron oxides are chemical compounds which have different polymorphic forms, including γ-Fe_2_O_3_ (maghemite), Fe_3_O_4_ (magnetite), and FeO (wustite). Among them, the most studied are γ-Fe_2_O_3_ and Fe_3_O_4_, as they possess extraordinary properties at the nanoscale (such as super paramagnetism, high specific surface area, biocompatible etc.), because at this size scale, the quantum effects affect matter behavior and optical, electrical and magnetic properties. Therefore, in the nanoscale, these materials become ideal for surface functionalization and modification in various applications such as separation techniques, magnetic sorting (cells and other biomolecules etc.), drug delivery, cancer hyperthermia, sensing etc., and also for increased surface area-to-volume ratio, which allows for excellent dispersibility in the solution form. The current methods used are partially and passively mixed reactants, and, thus, every reaction has a different proportion of all factors which causes further difficulties in reproducibility. Direct active and complete mixing and automated approaches could be solutions to this size- and shape-controlled synthesis, playing a key role in its exploitation for scientific or technological purposes. An ideal synthesis method should be able to allow reliable adjustment of parameters and control over the following: fluctuation in temperature; pH, stirring rate; particle distribution; size control; concentration; and control over nanoparticle shape and composition i.e., crystallinity, purity, and rapid screening. Iron oxide nanoparticle (IONP)-based available clinical applications are RNA/DNA extraction and detection of infectious bacteria and viruses. Such technologies are important at POC (point of care) diagnosis. IONPs can play a key role in these perspectives. Although there are various methods for synthesis of IONPs, one of the most crucial goals is to control size and properties with high reproducibility to accomplish successful applications. Using multiple characterization techniques to identify and confirm the oxide phase of iron can provide better characterization capability. It is very important to understand the in-depth IONP formation mechanism, enabling better control over parameters and overall reaction and, by extension, properties of IONPs. This work provides an in-depth overview of different properties, synthesis methods, and mechanisms of iron oxide nanoparticles (IONPs) formation, and the diverse range of their applications. Different characterization factors and strategies to confirm phase purity in the IONP synthesis field are reviewed. First, properties of IONPs and various synthesis routes with their merits and demerits are described. We also describe different synthesis strategies and formation mechanisms for IONPs such as for: wustite (FeO), hematite (α-Fe_2_O_3_), maghemite (ɤ-Fe_2_O_3_) and magnetite (Fe_3_O_4_). We also describe characterization of these nanoparticles and various applications in detail. In conclusion, we present a detailed overview on the properties, size-controlled synthesis, formation mechanisms and applications of IONPs.

## 1. Introduction

**Figure 1:**
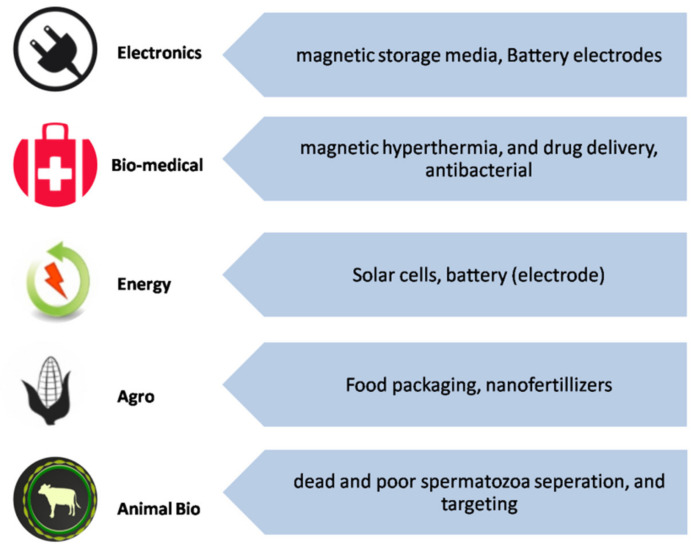
Various applications of iron oxide nanoparticles (IONPs).

Iron oxide nanoparticle (IONP)-based technologies are catalyzing rapid developments in nanotechnology. Due to technological importance, extensive research has been carried out on the development of various synthetic routes to yield IONPs with desired properties [1]. Among IONPs, mainly Fe_3_O_4_ and γ-Fe_2_O_3_ are extensively studied [2]. In general, iron oxides are classified into different phases (magnetite, hematite, maghemite, wustite). In the nano form, a material possesses interesting optical, magnetic, and electrical properties which cannot be found in their bulk form. This phenomenon can be described as the “quantum size effect” [3,4,5]. In the nanometer range of IONPs, the quantum effect dominates the behavior-affecting magnetic, electric, and optical properties of the matter. In the nanoscale, there is an impact of specific individual atoms or molecules, while in the bulk form, property is attributed to the average of all the quantum forces that affect all of the atoms. For example, magnetic Fe_3_O_4_ nanoparticles are superparamagnetic below the size of 20 nm [6]. As the nanoparticle size decreases, this property tends towards paramagnetic or superparamagnetic magnetization. Therefore, a decrease in nanoparticle size will enhance superparamagnetic behavior and decrease ferromagnetic behavior. As the size of nanoparticles decreases, the relative oxygen concentration decreases; therefore, a slight reduction in the iron valance state occurs. Because of this ferrous ion content increase, an increase in magnetization should also be observed [7]. Similarly, γ-Fe_2_O_3_ nanoparticles have gained technological importance due to their magnetic and catalytic properties. High magnetization and hysteretic heating make them potential candidates in separation and biomedical areas, and the semiconducting property and chemically active surface allow catalytic activities such as photocatalytic ability [8,9]. Iron oxide nanoparticles (IONPs) have a broad range of significant applications in electronics [10,11], biomedicine [12,13,14], energy [15,16], agriculture [17,18], and animal biotechnology [19,20], as shown in Figure 1. In a small size of about 10–20 nm, the superparamagnetic properties of Fe_3_O_4_ and γ-Fe_2_O_3_ nanoparticles become apparent, therefore, better performance can be achieved for the above-mentioned applications. Additionally, due to the increased surface-to-volume ratio, they show excellent dispensability in solutions [21].

**Figure 2:**
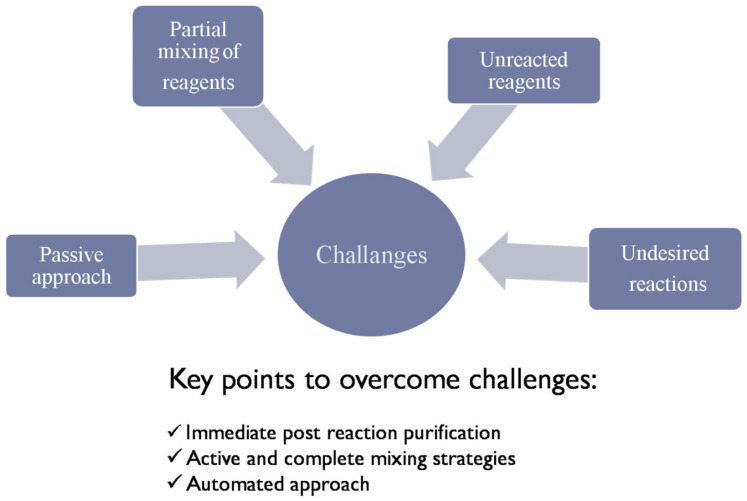
Challenges and key points in reproducible synthesis of nanoparticles.

However, reproducible synthesis of IONPs with desired properties is still a problem [22]. This is because existing synthesis methods show a passive approach towards synthesis reaction. The main challenges and key points to overcome them are explained in Figure 2. In existing methods, reactants are mixed partially and passively. Unreacted components therefore effect the final product when undesired reactions takes place, as the proportion of all these factors is different in every reaction, making it difficult to achieve reproducibility in the desired properties [23]. Immediate purification of nanoparticles after reaction becomes necessary to minimize error. Direct active and complete mixing of reactants and automated approaches could solve this issue. Researchers are mainly focused on size- and shape-controlled synthesis, as size determines the surface area, which plays a key role in its exploitation for scientific or technological purposes [24].

Manipulation of reaction parameters is necessary to obtain controlled nanoparticles in terms of size, shape, purity, crystallinity, and morphology. A synthetic route should enable control over reaction parameters: temperature; concentration; fluctuation in temperature; pH; stirring rate; particle distribution; size control; control over shape; nanoparticle composition and structure, which includes crystallinity, purity, rapid screening, and reliable adjustment of parameters [22,25,26,27].

In our opinion, the established synthetic routes of iron oxide nanoparticles have difficulty in controlling the particle size, shape, and properties. Many of the reported methods have their own pros and cons, as described in Table 1. It is necessary to develop a new synthetic route for IONPs that yields nanoparticles in a reproducible manner with excellent size control. This review explains various dimensions associated with synthesis of IONPs and their applications, and different synthesis mechanisms are summarized. Figures S1–S7 represented in Supplementary Materials corresponds to various IONPs synthesis methods graphically presented which also includes key points for each corresponding method.

## 2. Synthesis of IONPs

### 2.1. FeO Nanoparticle Synthesis

With ordered distribution of iron vacancies, wustite (FeO) is a defect rock salt structure that is metastable and anti-ferromagnetic, and it has a non-stoichiometric phase (mixed valency of Fe**^2+/3^**^+^) and p type semiconducting oxides with a high lattice defect concentration [49,50,51]. Changing the size and shape of FeO nanoparticles so as to manipulate the surface energy contribution is the best way to receive better stability for FeO nanoparticles. An alternative way would be to synthesize the core/shell (FeO/iron oxide) morphology by controlling oxidation.

**Figure 3:**
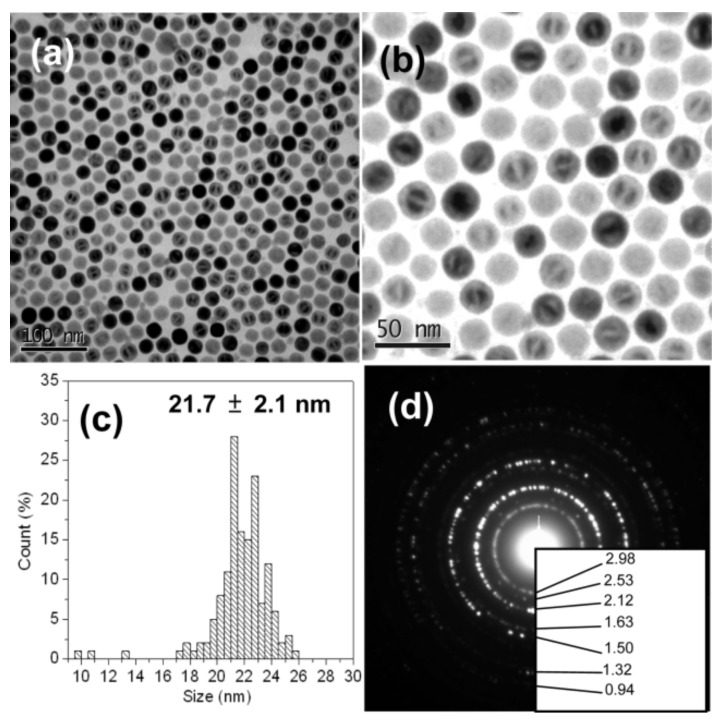
(**a**,**b**) TEM of FeO nanoparticles synthesized by thermal decomposition, (**c**) particle size distribution plot, (**d**) SAED pattern. Obtained permission from Ref. [53]. Copyright 2010 American Chemical society.

Strobel et al. [52] synthesized FeO, Fe_2_O_3_, and Fe_3_O_4_ nanoparticles by the physical method of flame spray pyrolysis mainly by controlling the fuel-to-air ratio during the combustion process. This also depends on the Fe valance state used in the precursor. While precursors Fe(III)-nitrate and Fe(II)-naphthenate are used in the synthesis, it is difficult to obtain a pure FeO phase; however, at a specific stoichiometric combustion, FeO formation is possible. Scalability and rapid synthesis of various nanostructured materials are the advantages, but it is an expensive method. Chen et al. [53] synthesized monodispersed FeO nanoparticles with a size of 21.7 ± 2.1 nm (Figure 3a,b) i.e., TEM image) by thermal decomposition of an Fe(III) oleate compound, using oleic acid as a solvent and at a high temperature of 380 °C. Figure 3c is the size distribution plot and Figure 3d is SAED pattern which shows mixed iron oxide phase corresponding to wustite and spinel type. In this work, the TEM device Philips Tecnai G2 F20 or JEOL JEM 2010 with an accelerating voltage of 200 kV was used, and Shimadzu XRD-6000 using a Cu Kα radiation device was used for XRD analysis. XRD analysis confirmed the FeO phase corresponding to the fcc oxygen sub lattice (rock salt-type structure). However, this is (fcc oxygen sub lattice) present in both wustite and spinel type iron oxides; therefore, it is difficult to make predictions based on the XRD pattern. Major peaks may have slight differences in their position [53,54]. 

**Figure 4:**
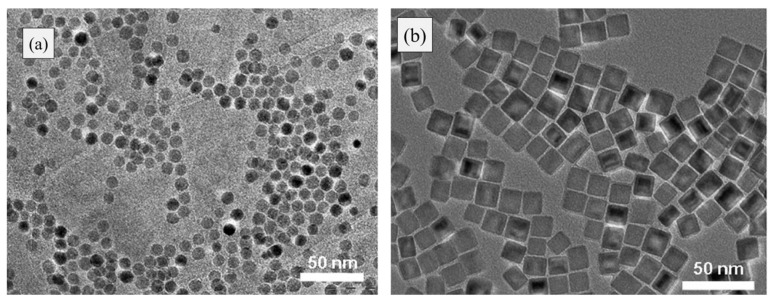
TEM micrographs: (**a**) spherical shaped and (**b**) cubical shaped. Obtained permission from Ref. [55]. Copyright 2011 American Chemical society.

There are less reports explaining the synthesis of the core/shell structure in which FeO, the core and shell (used in spintronics and recording media), is partially oxidized iron oxide. High coercivity may have great potential in the field of nanomagnets [51,55]. Pichon et al. [55] synthesized spherical- (12 ± 1.4 nm) and cubic- (13.2 ± 1.4 nm and 30.2 ± 2.4 nm) shaped nanoparticles (Figure 4a,b) by the thermal decomposition method. XRD and TEM analysis were conducted using the following devices: Bruker D8 Advance equipped with a monochromatic copper radiation source, (KR = 0.154056 nm) and a JEOL 2100F electron microscope working with a 200 kV accelerating voltage equipped with a GATAN GIF 200 electron imaging filter, respectively. Nucleation and growth dynamics affecting size and shape were tuned by controlling reaction parameters such as the heating rate and boiling point. XRD analysis shows the presence of the wustite phase and spinel phase. TEM analysis shows the wustite core and spinel shell for cubical nanoparticles with a size of 5 ± 1 nm and 17 ± 1 nm, and for spherical nanoparticles with a size of 20 ± 1 nm and 30 ± 1 nm, respectively. 

Khurshid et al. [51] synthesized partially oxidized FeO nanoparticles to generate an FeO core and a magnetite (Fe_3_O_4_) shell by thermal decomposition of iron-organometallic salts at a high temperature. Size is inversely proportional to an increase in heating rate and directly proportional to boiling temperature. Deconvoluted XRD spectra into constituent peaks show prominent peaks matching with the FeO phase. However, the coexistence of the Fe_3_O_4_ phase with the FeO phase, which might be due to surface oxidation of FeO forming a core/shell structure, is also confirmed by the SAD (selected area diffraction) pattern. Glaria et al. [54] synthesized FeO nanoparticles (~5 nm) with controlled hydrolysis of an organometallic precursor at room temperature. The magnetic property observations of Fe^3+^/Fe^2+^at the surface show that ferromagnetic/antiferromagnetic bonds decrease from the shell to the core.

**Figure 5:**
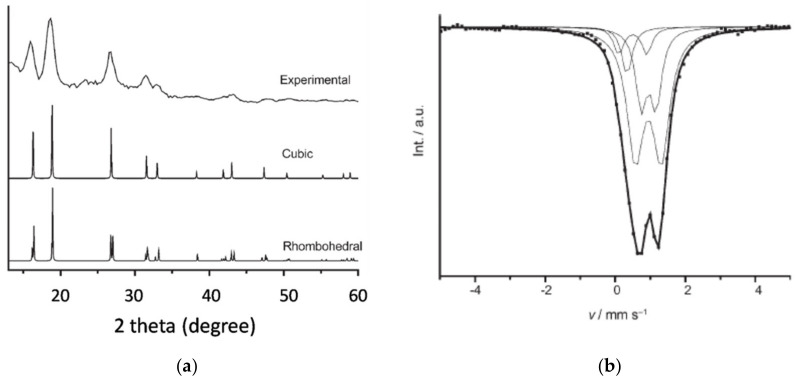
(**a**) XRD pattern of FeO nanoparticles: (**b**) Mössbauer spectrum of FeO nanoparticles at room temperature. Obtained permission from Ref. [54]. Copyright 2008 John Wiley and Sons.

The XRD spectrum was obtained by the sample irradiated with molybdenum Ka radiation (0.71069 K) with a dedicated diffractometer. The Mössbauer spectrum was obtained by a Wissel MR360 transducer (a Wissel DFG1000 function generator and a Canberra meter with 25 mCi^57^Co source embedded in rhodium matrix). XRD patterns of FeO nanoparticles with peaks of experimental data, and cubic and rhombohedral phases are presented in Figure 5a.There is no significant difference in the peak positions for the cubic and rhombohedral phases; however, broadening of peaks due to small sized nanoparticles makes phase identification difficult. However it is possible to differentiate different FeO phase on the basis of cell parameters in XRD analysis [54,56].The asymmetrical broad doublet corresponding to nonstoichiometric FeO formation confirmed by Mössbauer measurements was carried out in zero field, as shown in Figure 5b, and it can be performed more accurately when the sample is not exposed to air. Mössbauer measurements are useful to study the magnetic structure and the phase state of the synthesized IONPs, and it is a much more sensitive technique than XRD. In the case of IONP analysis, the doublet in Mössbauer measurements signifies that the matrix consists of other atoms around Fe atoms or that the Fe atoms are not magnetically coupled. It also indicates oxide formation at the surface [49,50,54].

### 2.2. α-Fe_2_O_3_ Nanoparticle Synthesis

Akbar et al. synthesized α-Fe_2_O_3_ nanoparticles with the sol–gel method, with size ranging from 22 to 56 nm by controlling various reaction parameters such as concentration, annealing temperature etc. Size was found to be directly proportional to concentration while inversely proportional to annealing temperature [57]. The XRD study was carried out by using X-ray diffractometer (JDX-11 Jeol), and Mössbauer spectroscopy was carried to explore magnetic identification of the particles. Although XRD patterns of samples (mixture of α-Fe_2_O_3_ and γ-Fe_2_O_3_) prepared at different annealing temperatures showed a difference, an attempt was made to identify the phase by using a relative ratio of two major peaks. The best-fitted Mössbauer spectra confirmed the presence of α-Fe_2_O_3_ and γ-Fe_2_O_3_ phases. Morales et al. [58] prepared α-Fe_2_O_3_ nanoparticles by the controlled precipitation method with a size of less than 100 nm, but the main drawbacks were aggregation and phase impurity. Phase transformation occurred at a high temperature, i.e., from the γ phase to α phase. It seems very difficult to specifically characterize Fe_2_O_3_ phases in XRD(XRD device used was Philips PW1710 BASED X-ray Diffractometer with 2θ values from 10° to 89.98° with a Cu Kα radiation source (λ = 1.54274) operating at 38 kV and 23 mA), and the presence of a magnetite phase cannot be ignored because of surface oxidation and the similarity between diffraction lines [58,59,60,61]. However, iron oxide phases can be identified in better way, that is, with combined Mössbauer and Raman spectroscopy [57,58]. 

Sarangi et al. [59] reported a cost-effective method to synthesize single-phase α-Fe_2_O_3_ nanoparticles with size of about 20–30 nm. Nanoparticles were obtained by a calcinating precursor (ferric nitrate nonahydrate) at high temperature in air. The formation of single phase α-Fe_2_O_3_was confirmed by XRD, TEM and SEM. XRD, TEM and SEM characterization were performed by using a powder X-ray diffractometer with Cu Kα radiation in the 2θ range of 20° to 70°, with a scan rate of 2°/min (Mini Flex II, Rigaku, Japan), FEI HRTEM (Tecnai G2 30S Twin) at an accelerating voltage of 300 kV, and JEOL (JSM 6560 LV) SEM at accelerating voltage of 20 kV, respectively. Thermogravimetric analysis was carried out to analyze the decomposition behavior of precursor.

**Figure 6:**
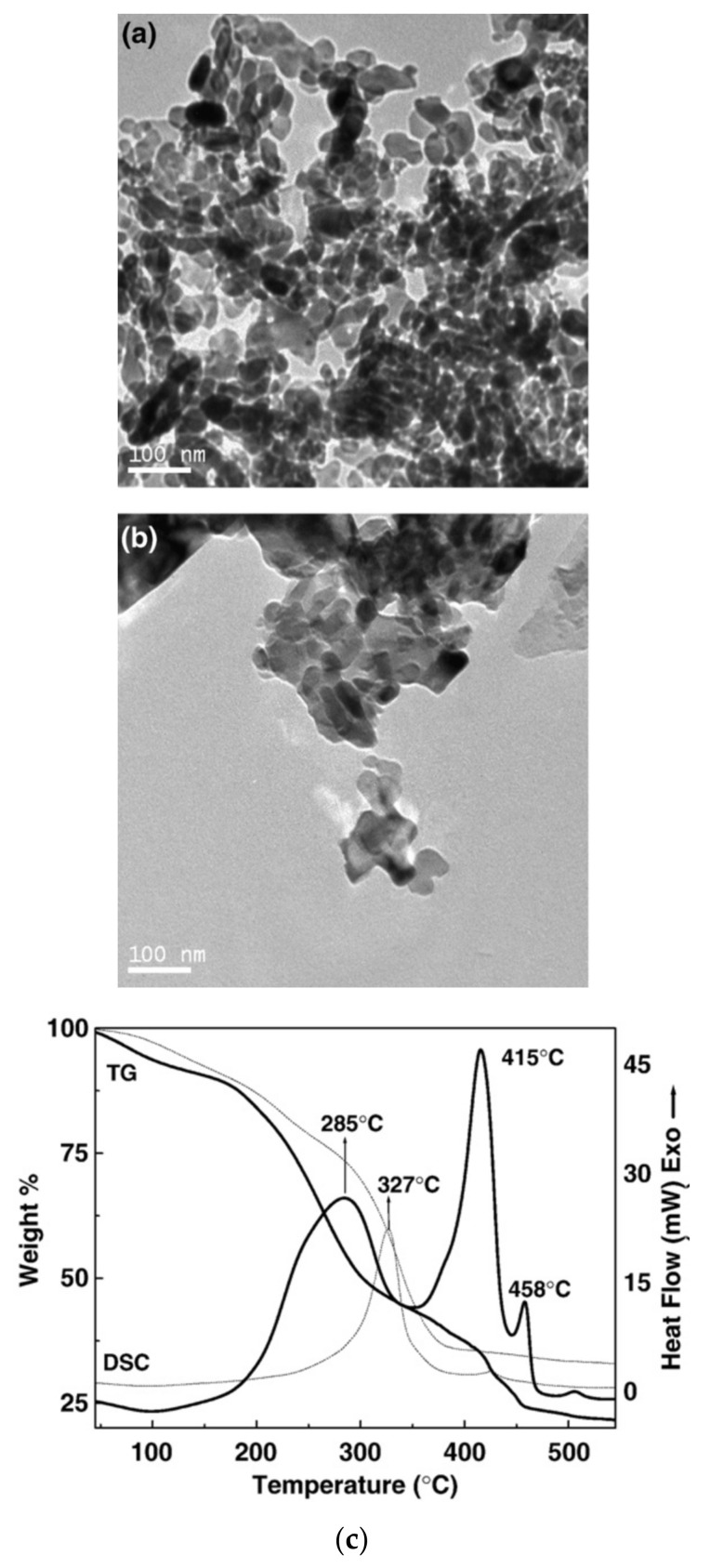
(**a**,**b**)TEM images of α-Fe_2_O_3_ nanoparticles calcinated at 450 °C, (**c**) thermogravimetric analysis of precursors in air. Obtained permission from Ref. [59]. Copyright 2009 Elsevier.

In thermogravimetric analysis, major weight loss (48%, 20% and 51%) at temperatures 140 to 350 °C, 355 to 460 °C and 200 to 450 °C, respectively, were attributed to the decomposition of the precursor and formation of α-Fe_2_O_3_. Figure 6 shows TEM images of α-Fe_2_O_3_ nanoparticles prepared by a calcinating precursor at 450 °C. Due to the fact that beyond 460 °C there is no new peak or weight loss occurrence, it is confirmed that at this temperature, full decomposition of the precursor to iron oxide occurs.

**Figure 7:**
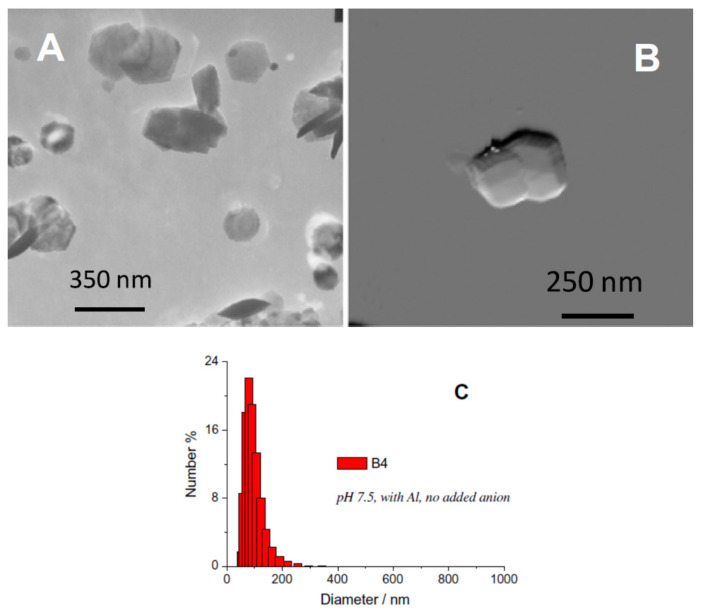
(**A**,**B**) TEM and AFM images of typical rhombohedral shape of α-Fe_2_O_3_ nanoparticles, respectively; (**C**) size distribution obtained by dynamic light scattering(DLS). Obtained permission from Ref. [60]. Copyright 2012 Elsevier.

Colombo et al. [60] synthesized pure phase α-Fe_2_O_3_ nanoparticles by a multi-step approach in which dehydration of ferrihydrite was carried out in specific conditions. Hydrolysis of Fe(NO_3_)_3_was carried out with KOH in the presence of different additives such as organic acids with low molecular weight and at different temperatures and concentrations. Additives act as templates that affect surface area and particle size, modifying the crystallochemical nature of the hematite growth. The typical rhombohedral shape of α-Fe_2_O_3_ nanoparticles can be seen in Figure 7 with a mean particle diameter of 92 nm. This work uses TEM in combination with AFM (atomic force microscopy) and highlights that in order to understand α-Fe_2_O_3_ particle geometry, formation and aggregation mechanisms with combined AFM and DLS (dynamic light scattering) experiments can be useful. TEM analysis was carried out by using Jeol JEM-200CX operating at 200 kV, and for AFM analysis, a Nano-Scope III atomic force microscope with a Multiplode SPM unit (Digital Instrument, Inc., New York, NY, USA) was used.

Figure 7A shows a TEM image ofα-Fe_2_O_3_ nanoparticles prepared by aging of ferrihydrite at 371 K and at a pH of 7.5 in the size range of 70 nm to 135 nm. AFM analysis also provides roughness, height and particle size. In this work, data on vertical height were recorded digitally so as to obtain surface topography; these data were used to estimate the surface roughness. Surface roughness (Ra) was estimated with Nanoscope IIIa Software version 4.22r2 (Digital Instrument, Inc., New York, NY, USA), which was calculated as root mean square of the surface height measurement. All these results were obtained from analysis of 20 nanoparticles of hematite (α-Fe_2_O_3_). The AFM micrograph (Figure 7B) shows a marginally higher size of about 200 nm and 100 nm. The average surface calculated was 0.16 nm. It was also observed that particle size analysis conducted by AFM and TEM was quantitatively comparable. The DLS (Figure 7C) study confirms the presence of monodispersed smaller crystals with a mean peak diameter of 92 nm.

However, although there are reports on α-Fe_2_O_3_synthesis, one of the key problems is aggregation, and a lot of work on surface reactivity is required so as to understand the aggregation mechanism.

### 2.3. γ-Fe_2_O_3_ Nanoparticle Synthesis

Girod et al. [62] synthesized γ-Fe_2_O_3_ nanoparticles by the co-precipitation method at different temperatures. At low temperatures, particles tend to aggregate, and size is inversely proportional with temperature. At 30 °C, around 30 nm particles were synthesized with aggregation, while at 80 °C, around 7 nm particles without aggregation were synthesized. No aggregation at high temperature can be speculated due to directed crystallization. In short, it can be speculated that high temperature may affect purity, providing a higher percentage of purity as compared to lower temperatures. Hence, temperature is a very important factor affecting size and stability.

**Figure 8:**
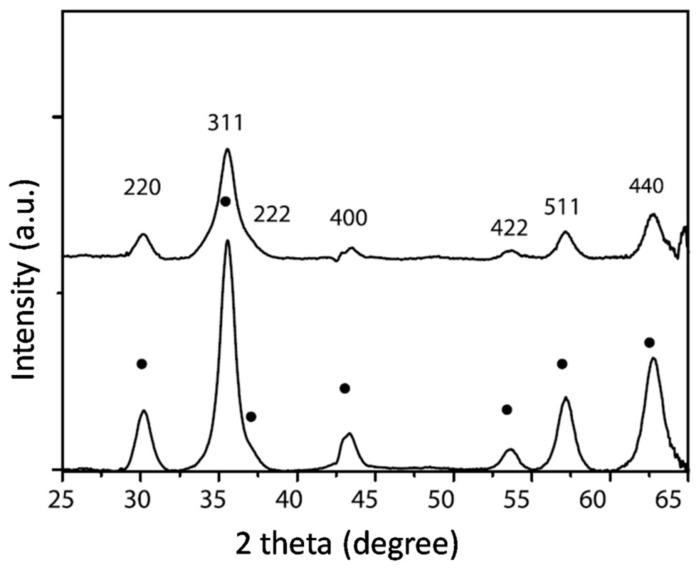
XRD pattern of iron oxide nanoparticles (top: 30 °C and bottom: 80 °C); γ-Fe_2_O_3_ phase indicated by black dots. Obtained permission from Ref. [62]. Copyright 2015 Elsevier.

Figure 8 shows the XRD pattern (XRD analysis was carried out with a μ-Spot beam line at BESSYII Berlin, Germany) of iron oxide nanoparticles where black dots represent γ-Fe_2_O_3_ nanoparticles at different temperatures (top: 30 °C and bottom: 80 °C). The crystallite diameter calculated using (311) reflection was 6.8 nm and 5.83 nm for particles synthesized at 80 °C and 30 °C, respectively. However, the main drawback is that XRD analysis lacks the ability to distinguish among iron oxides as they have similar patterns, as mentioned earlier [57,58,59,60,61,62]. Nevertheless, in many reports, attempts are made to identify γ-Fe_2_O_3_ with XRD by denoting (220), (311), (400), (422), (511) and (440) planes [62,63,64].

Parallel studies have been reported by researchers, such as-solid state reaction synthesis [64], co-precipitation using nitric acid [65], and oxidation of magnetite nanoparticles prepared by co-precipitation [66].

Nurdin et al. [67] synthesized γ-Fe_2_O_3_ nanoparticles at different temperatures and concentrations with size ranging from around 9 nm to 17 nm with spherical morphology by the co-precipitation method. The crystalline γ-Fe_2_O_3_ phase was confirmed by XRD. XRD analysis was carried out by Philips X’PertMPD X-Ray Diffractometer, using a copper source (λ = 1.54056 ˚A) with a scan range of 20–80°, a 2θ angle at a step of 0.05° and a count time of 5 s at each step. TEM images were taken by using a Leo LIBRA transmission electron microscope operated at 120 kV.

**Figure 9:**
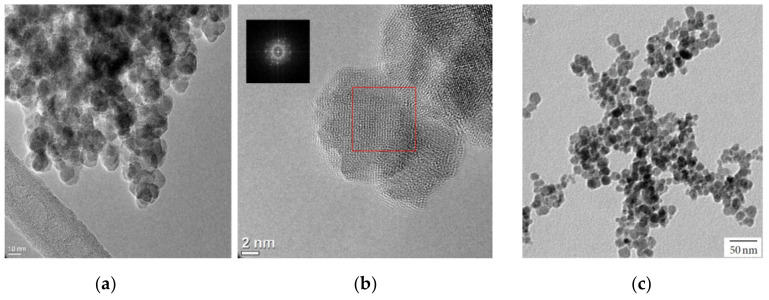
TEM: γ-Fe_2_O_3_ nanoparticles, (**a**,**b**), (**c**). Obtained permission from Ref. [67]. Copyright 2015 Elsevier, Obtained permission from Ref. [68]. Copyright 2014 Hindawi Publishing Corporation.

Spherical-shaped γ-Fe_2_O_3_ nanoparticles show a particle size of 15.6 ± 2.68 nm, which is in good agreement with the crystalline size of 13.9 ± 1.15 nm; this indicates that nanoparticles are monocrystals, which is denoted in Figure 9c. By controlling nitric acid concentration in a co-precipitation method, size was controlled. In another report, Ramos et al. [68] synthesizedγ-Fe_2_O_3_ nanoparticles with a spherical size of around 10 nm by oxidation of magnetite, as shown in Figure 9a; HRTEM micrograph (using transmission electron microscopy (TEM) (Titan 80300 Kv) is shown in Figure 9b, and the corresponding electron diffraction pattern reveals a spinel type structure of maghemite. Heat treatment was given to magnetite nanoparticles synthesized by the co-precipitation method. Tural et al. [69] and Staraha et al. [61] synthesized γ-Fe_2_O_3_/SiO_2_ (9 ± 2 nm) and γ-Fe_2_O_3_/Au (12.2 ± 1.9 nm) nanocomposites by sol–gel and precipitation methods, respectively.

### 2.4. Fe_3_O_4_ Nanoparticle Synthesis

Physical and chemical properties of Fe_3_O_4_ nanoparticles and their surface also have effects on the performance of these nanoparticles. Some new methods for the synthesis of Fe_3_O_4_ nanoparticles using thermal degradation of an iron precursor at high temperature have been developed, but this type of method needs very high temperature and is complicated. In the thermal decomposition method, the following are the main factors: the presence of a suitable organo-metallic precursor that decomposes below the surfactant degradation temperature, two surfactants which can differentially be adsorbed on nanocrystal faces, and one surfactant must promote the monomer exchange between particles to achieve size distribution [70,71,72].There are stringent requirements for the application of Fe_3_O_4_ nanoparticles in bio-medical science, including: (a) Size should be smaller than 20 nm [73,74] for easy penetration and motion. (b) The surface should be uncapped, thereby allowing the binding of a therapeutic molecule. (c) As the size decreases, T_c_ (critical transition temperature) decreases. Therefore, magnetic nanoparticles should be used below T_c_ so as to utilize their magnetic property. Thus, a correct size of the nanoparticles having required the critical transition temperature (T_c_), is critical for their desired application. (d) Finally, synthesis should be highly reproducible, scalable, and low cost. Recently, there are also so many advancements in the synthesis of Fe_3_O_4_ nanoparticles by using colloidal chemical approaches [22].

Chemical methods (using either aqueous or organic solutions) and the polyol method (the method of reducing metallic oxide or the metallic complex compound by polyol in an organic solvent) are mainly employed for the synthesis of Fe_3_O_4_ nanoparticles. Table 2 explains some limitations in co-precipitation and the polyol method.

Magnetite nanoparticles were synthesized by the hydrolysis of Fe^2+^ ion and Fe^3+^ ion (mole ratio of 1:2) by a base (e.g., NaOH, KOH, NH_4_OH) [75,76,77]. During this, the overall composition of the precipitate was the same as that of the reaction system. However, since the hydrolysis rate of Fe^3+^ ions is greatly different from that of Fe^2+^ ions, the composition of the nanoparticle may not be the same [22,27]. At pH > 11, re-dissolutions of Fe(OH)_3_ and Fe(OH)_2_ begins:

Fe(OH)_3_ → Fe(OH)_4_^−^Fe(OH)_2_ → Fe(OH)_3_^−^

Additionally, in the polyol method the origin of oxygen in magnetite is still unclear; therefore, the mechanism leading to Fe_3_O_4_ formation in the polyol method is not fully understood.

Thus, when the magnetite particles (Fe_3_O_4_) that have a particular stoichiometric composition are made by the co-precipitation method, the pH adjustment or pH control is a very important and tedious task. There are some other disadvantages of the co-precipitation method, such as broad nanoparticle size distribution, poor crystallization and irregular crystal shape [22,27]. The polyol method has its own disadvantages, for example, it requires along synthesis time (minimum 7–8 h) and high temperature (>200 °C) [22]. The exact mechanism leading to the formation of Fe_3_O_4_ and the origin of the oxygen element in Fe_3_O_4_ is still unclear in the polyol method. T. Daou et al. reported Fe_3_O_4_ nanoparticle synthesis with a size of 39 nm ± 5 nm by hydrothermal reaction of 1 M ferric chloride hexa-hydrate (FeCl_3_·6H_2_O) and 2 M ferrouschloride tetra hydrate (FeCl_2_·4H_2_O) dissolved in 2 M HCl followed by slow addition of aqueous solution of tetramethyl ammonium hydroxide [N(CH_3_)_4_OH]. TEM and HRTEM images were obtained with a TOPCON model 002B transmission electron microscope operating at 200 kV and at a point-to-point resolution of 0.18 nm.

At 70 °C, co-precipitation was carried out from Fe^2+^ and Fe^3+^ ions by [N(CH_3_)_4_OH], and at 250 °C hydrothermal treatment was carried out. This is a very slow process and requires high temperature [78]. In the work of Mascolo et al. [79], 63 nm ± 25nm Fe_3_O_4_ nanoparticles were synthesized by the co-precipitation method in alkaline condition using FeCl_3_ and FeCl_2_ salts. TEM analysis was carried out by using the device FEI Tecnai G12 Spirit Twin, Hillsboro, OR, USA. Initially, ferrous and ferric hydroxides were prepared rapidly. Ferric hydroxide decomposes to FeOOH. Finally FeOOH and Fe(OH)_2_ form magnetite by solid state reactions. A drawback of this method is the large particle size as it is not suitable for medical applications. Panta et al. [80] synthesized oleic acid- and PEG(poly ethylene glycol)- (as a surfactant agent) coated Fe_3_O_4_ nanoparticles by the chemical co-precipitation method.

Several other methods have been reported for the synthesis of Fe_3_O_4_ nanoparticles, such as chemical methods using plant extracts [81,82] and bacteria [83,84] as reducing agents, thermal decomposition/pyrolysis of organo-metallic precursors [85,86], ultrasound irradiation [87], gamma radiolysis [88], thesol–gel method [89] etc. Most of these methods yield polydisperse nanoparticles, surface capped nanoparticles and nanoparticles with impurities, in addition to poor reproducibility. Different polymers and surfactants such a polyvinylalcohol (PVA) [90], poly(vinylpyrrolidone) (PVP) [91], polyethylene glycol (PEG) [92], oleic acid [93] and polyacrylic acid (PAA) [94] are used for the coating of Fe_3_O_4_ nanoparticles or as capping agents (for size control during the synthesis and suppression of the aggregation). Capping results in improved morphology, prevention of agglomeration and aggregation, but may affect the properties of nanoparticles. Furthermore, polymers and surfactants are expensive and difficult to (naturally) decompose. Thus, their use restricts the applications of Fe_3_O_4_ nanoparticles in biomedical science and also can cause environmental problems. Nene et al. [22,26,27] developed a new method to synthesize Fe_3_O_4_ nanoparticles by ascorbic acid-mediated reduction of Fe(acac)_3_. This method allows for size-controlled synthesis with reproducibility. TEM image were obtained from a JEOL JEM-2100F (USA) microscope.

**Figure 10:**
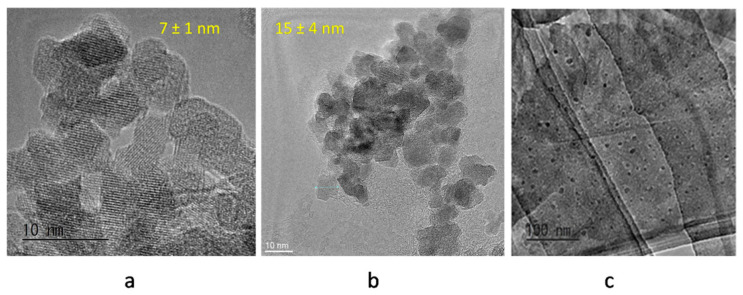
Fe_3_O_4_ nanoparticles synthesized by ascorbic acid mediated reduction of Fe(acac)_3_, (**a**) without water, (**b**) with water, (**c**) graphene–Fe_3_O_4_ nanoparticles [22,25,26,27].

Synthesis reaction was carried out in the presence (Figure 10b) and in absence of ultrapure water (Figure 10a) to examine the role of water and thus to understand the mechanism of formation clearly. Moreover, this method was extended to synthesize a graphene–Fe_3_O_4_ nanohybrid composite (Figure 10c) [25].The mechanism of magnetite formation is much more clearly explained in these reports, and the role of water is explored. It was found that in the absence of water, Fe/iron oxide nanoparticles were generated with a size of 7 ± 1 nm. Table 3 displays reagents used in various IONP synthesis methods with their used quantity. The concentrations in the second column correspond to the number of moles, i.e., molar (M) or millimolar (mM), weight in grams (g), percentage (%).

## 3. Different Mechanisms of Iron Oxide Nanoparticle Synthesis

In this section, the mechanisms of IONP formation involved in different synthetic routes are explained. The co-precipitation method and the hydrothermal method are widely used on a commercial scale to synthesize different IONPs. By controlling reaction parameters such as concentration of reactants, pH, temperature, nucleation etc., the phase of oxides, size, and shape can be controlled. Most commonly, the co-precipitation method is widely used for synthesizing IONPs due to its simplicity, where pH control remains as a main but tedious task to achieve.

Solid State Reactions of Fe_2_O_3_ nanoparticle formation can be given as follows:

FeCl_2_ + 2FeCl_3_ + 8NaOH → Fe_3_O_4_ + 8NaCl + 12H_2_O

In the above reaction system, FeCl_2_ and FeCl_3_ act as Fe precursors giving Fe^2+^ and Fe^3+^ ions. NaOH is the base and acts as oxygen or the OH supplier. Instead of NaOH, other bases such as KOH are also used. In this step, Fe(OH)_2_ and Fe(OH)_3_ are formed after the reaction with the base generating an initial precursor Fe_3_(OH)_8_. After hydrolysis, Fe_3_O_4_ nanoparticles are generated [64].

4Fe_3_O_4_ + O_2_ → 6γ-Fe_2_O_3_ (Maghemite)

γ-Fe_2_O_3_ → α-Fe_2_O_3_(Hematite)

The mechanism of the formation of γ-Fe_2_O_3_ by the decomposition of magnetite using HNO_3_ as an oxidizing agent and can be given as [67]:

2FeCl_3_ + FeCl_2_ + 8NH_4_OH → Fe_3_O_4_ + 4H_2_O + 8NH_4_Cl

2Fe_3_O_4_ + HNO_3_ → 3γ-Fe_2_O_3_ + HNO_2_

In above method, Fe_3_O_4_ nanoparticles were synthesized by the co-precipitation method, and 3γ-Fe_2_O_3_ were obtained upon their decomposition.

Mascolo et al. [79] synthesized Fe_3_O_4_ nanoparticles under inert nitrogen atmosphere by Co-Precipitation reaction at room temperature and in the presence of bases such as NaOH, KOH or (C_2_H_5_)_4_NOH:

2FeCl_3_ + FeCl_2_ + 8 BOH → Fe_3_O_4_ + 4H_2_O + BCl

In the above equation B = Na^+^, K^+^ or (C_2_H_5_)_4_N^+^. The following equations are proposed in this report for Fe_3_O_4_ formation.

Fe^3+^ + 3OH → Fe(OH)_3_

Fe(OH)_3_ → FeOOH + H_2_O

Fe^2+^ + 2OH^−^ → Fe(OH)_2_

2FeOOH + Fe(OH)_2_ → Fe_3_O_4_ + 2H_2_O

Overall, the reaction equation can be given as:

2Fe^3+^ + Fe^2+^+ 8OH^−^ → 2Fe(OH)_3_Fe(OH)_2_ → Fe_3_O_4_ + 4H_2_O

In this method, FeCl_2_·4H_2_O and FeCl_3_·6H_2_O were dissolved in degassed water, which was followed by the addition of NaOH at room temperature [86,95]. This is a one pot direct synthesis method. The molar ratio of 2:1 (ferric to ferrous) was designed to synthesize Fe_3_O_4_ nanoparticles particularly. The size of nanoparticles can be controlled by concentration, pH, the rate of mixing of reactants, and temperature.

Roonasi et al. [96] studied the mechanism of Fe_3_O_4_ formation using the Isotope Fractionation study.In this report, Fe(II) and Fe(III) alkali solutions were used. Synthesis of magnetite nanoparticles was performedby Co-Precipitation of Iron (II) and (III) and oxidation of Ferrous Hydroxide.

The overall reaction is as follows:

Fe^2+^ + 2Fe^3+^ +8OH^−^→Fe_3_O_4_ + 4H_2_O

Initially, precipitation of Fe(OH)_2_ occurs after the addition of an iron (II) chloride solution to sodium hydroxide solution as follows:

FeCl_2_ + 2NaOH → Fe(OH)_2_ + 2NaCl

Magnetite formation starts then with the oxidation of Fe(OH)^+^ in aqueous solution.

Fe (OH)_2(solid)_ [Fe (OH)]^+^_(aq)_ + OH^−^

Then, oxidation takes place as follows:

2[Fe(OH)]^+^_(aqueous)_ + ½ O_2_ + H_2_O → [Fe_2_(OH)_3_]^3+^_(aqueous)_ + OH

[Fe_2_ (OH)_3_]^3+^ then combines with [Fe (OH)]^+^. This lead to the formation of Fe_3_O(OH)_4_^2+^ having the same FeII/FeIII ratio as magnetite. Then, the following equation shows how to intermediate the transfer to crystalline magnetite.

Fe_3_O(OH)_4_^2+^_(aq)_ + 2OH^−^ → Fe_3_O_4(solid)_ + 3H_2_O

With the help of the isotope fractionation, the mechanism of Fe_3_O_4_ formation was studied in detail, and it was found that co-precipitation did not lead to the partition of the iron isotope which results in the Fe_3_O_4_ phase.

Qiaojuan et al. [97] synthesized biocompatible Fe_3_O_4_ nanocrystals by Pyrolysis of Fe(acac)_3_:
*HOOC-PEG-COOH* *(Polyethylene glycol)*+*2R-NH_2_* *(Oleamine)*→*RNH_3_^+−^OOC-PEG-COO^−+^H_3_NR* *(Amine salt)*
+*Fe(acac)_3_*
↓
*Fe_3_O_4_* NPs

In this method, in the presence of α, ω-dicarboxyl-terminated polyethylene glycol (HOOC-PEG-COOH) and oleylamine, the pyrolysis of Fe(acac)_3_ in diphenyl oxide was carried out to synthesize Fe_3_O_4_ nanocrystals. Polyethylene glycol (HOOC-PEG-COOH) can form a primary amine salt with oleylamine by donating its proton, leading to the formation of OOC-PEG-COO, which then coordinates with Fe(acac)_3_ by partly replacing the acetylacetonate ligand of Fe(acac)_3_. The carboxylated PEG first reacts with oleylamine forming the primary amine salt, which subsequently coordinates with the Fe atom in Fe(acac)_3_ by partly replacing the acetylacetonate ligand, consequently leading to the formation of a large molecular network. This molecular network then partly breaks down and forms the Fe_3_O_4_ nuclei. As the reaction progresses, Fe_3_O_4_ are nanocrystals synthesized. The size of the nanoparticles was controlled by gelification degree of stock solution and reaction time. This method shows the capabilities for adaption to regulate size and shape control of nanoparticles.

Xi et al. [98] synthesized rod-shaped Fe_3_O_4_ nanocrystals by the Poly (Vinyl Pyrrolidone) (PVP)-mediated hydrothermal synthesis method. Synthesis was carried out at 90 °C. In this mechanism, NaOH acts as s pH regulator and provides OH Ions, and KNO_3_ acts as an oxidizing agent. Initially, Fe(OH)_2_ is formed, and part of Fe(OH)_2_ is oxidized to Fe(OH)_3_.

The formation of Fe_3_O_4_ can be explained as follows:

Fe(OH)_2_ + 2Fe(OH)_3_ → Fe_3_O_4_ + 4H_2_O

3Fe(OH)_2_ + NO_3_^−^ → Fe_3_O_4_ + NO_2_^−^ + 3H_2_O

Abedini et al. [88] synthesized colloidal Fe_3_O_4_ nanoparticles by using the gamma radiolysis method. An aqueous solution containing FeCl_3_·6H_2_O, PVA (Poly Vinyl Alcohol) and isopropanol was prepared. pH of this solution was increased to 11 by adding NaOH solution at 70 °C. The final solution was irradiated by Gamma Rays.

H_2_O---irradiation → e^−^, H_2_, OH, H_2_O_2_ etc. form

Fe^3+^ +e^−^ → Fe^2+^

Fe^2+^+ 2Fe^3+^ + 8OH^−^ → Fe_3_O_4_ + 4H_2_O

The above equations show the formation mechanism for Fe_3_O_4_ nanoparticles. In this mechanism, PVA acts as stabilizer, and isopropanol and NaOH act as hydroxyl ion scavengers and stabilizers to control growth.

Nene et al. [22,26,27] reported Fe_3_O_4_ nanoparticle synthesis by reduction of Fe(acac)_3_ by Ascorbic acid to generate Fe_3_O_4_ nanoparticles. This method is similar to the Sol–Gel method. Fe(acac)_3_ (Fe(III) Acetylacetonate Compound) in diphenylether was reduced by Ascorbic acid and hydrolyzed using water. This is a much clearer mechanism of Fe_3_O_4_ formation in which water acts as source of Oxygen in magnetite and Ascorbic Acid acts as reducing agent and, therefore, a potential method for commercial scale, enabling reproducible size-controlled synthesis of IONPs. In short, Fe(acac)_3_ (Fe(III) Acetylacetonate Compound) in Diphenylether was reduced by Ascorbic acid and hydrolyzed using water. Fe(III) ion is reduced by the ascorbic acid at 60 °C and Fe(II)Fe(III)_2_(OH)_x_ (Precursor) is synthesized. Then, the reaction system is heated at 190 °C several times (30, 60, 90 min). This heating process (annealing) is very important as it is the crystallization process of precursor particles

The mechanism of Fe_3_O_4_ formation can be given as:

6Fe(III)(acac)_3_ + ascorbic acid (act as reducer) + 8H_2_O → 2Fe_3_O_4_ + dehydroascorbic acid + 18acac

**Figure 11:**
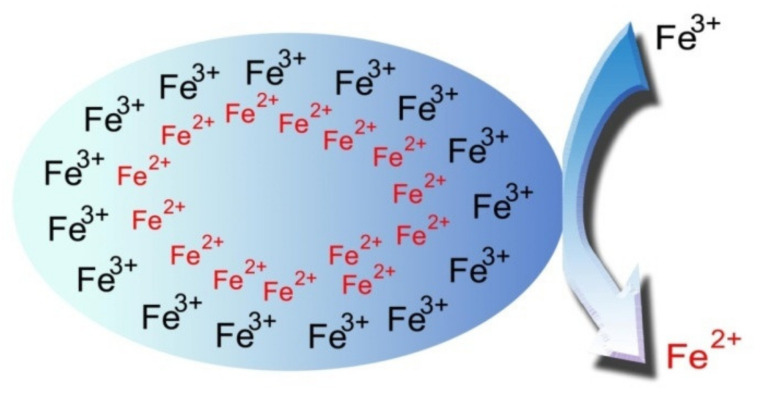
Droplet of ascorbic acid solution.

Ascorbic acid reduces the Fe(acac)_3_ as follows: 
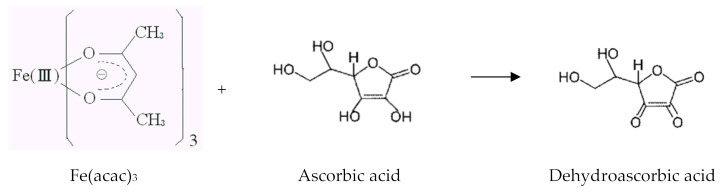


It is speculated (Figure 11) that when the ascorbic acid solution is added to the Fe(acac)_3_ diphenyl ether solution, small droplets containing ethanol, water and ascorbic acid are formed on the surface. Here, we consider the possibility that Fe^3+^ exists near the surface of droplet, while Fe^2+^ exists inside the droplet, as shown in Figure 11.

Fe^2+^ is formed because of the reduction of Fe^3+^(acac)_3_ by ascorbic acid, and because ultrapure water Fe (OH)_2_ is generated as follows:

Fe^3+^(acac)_3_ + ½ (C_6_H_8_O_6_) → Fe^2+^

2H_2_O <=>2H^+^+ 2(OH)^−^

Fe^2+^+ 2(OH)^−^ → Fe(OH)_2_

2Fe(OH)_3_ is formed as follows:

6H_2_O <=>6H^+^ + 6(OH)^−^

2Fe(acac)_3_ + 6(OH)^−^ + 6H^+^ → 2Fe(OH)_3_

In general, Fe(acac)_3_ is reduced by ascorbic acid, and Fe_3_(OH)_8_ is synthesized as follows:

2Fe^3+^ + Fe^2+^ + 8H_2_O → Fe_2_^3+^Fe^2+^(OH)_8_

When the reaction mixture is heated to reflux, it results in crystallization of Fe_3_O_4_ nanoparticles and hydrolysis. The formation of Fe_3_O_4_ is as follows:

Fe_3_(OH)_8_ → Fe_3_O_4_ + 4H_2_O

The general reaction can be written as:

6Fe(acac)_3_ + C_6_H_8_O_6_+ 8H_2_O → 2Fe_3_O_4_ +C_6_H_6_O_6_+18(acac)

## 4. Application of IONPs

The application of IONPs in biological science is characterized by the method of coating their surface with different inorganic and organic coatings. Magnetic NPs can be coated/encapsulated by nucleotide, proteins, antibodies, drugs, nonionic detergents, surfactants etc. [99]. In order to understand the wide range of applications, the number of disciplines is enlisted in the Table 4.

### 4.1. Biomedical Application

As below-30 nm IONPs are super-paramagnetic in nature and ideal for biomedical application, and because super-paramagnetic IONPs can be manipulated by external magnetic fields, with all these properties, the biomedical application of IONPs, such as in magnetic resonance imaging (MRI), drug delivery and hyperthermia, is possible [121].

#### 4.1.1. Biosensing Application of IONPs as Nanozymes

Recently there have been constructions of novel enzyme mimetics (nanozymes). These nanozymes showed the peroxidase catalase, superoxide-like activities and biological oxidase [122]. These nanozymes are a development in nanotechnology and possess intrinsic biological enzyme-like properties to offer advantages over natural enzymes. The examples of such advantage are high stability, cost effectiveness, easy manipulation, multiple applications in a single platform and activity under varying conditions such as temperature and pH. With an easily modulated shape and size, they are significantly more useful than natural enzymes. The enzyme-mimetic activities are used for the construction of non-enzymatic biosensors which can test the levels and concentrations of cholesterol, urea, glutathione, glucose, H_2_O_2_, biomarkers for cancer diagnosis and creatinine [123,124,125,126,127]. Some metal oxide-based nanomaterials (IONPs, silver, copper, gold) and nanosheets of graphene showed horseradish peroxidase (HRP)-like activity. It is still necessary to develop novel ways to increase the catalytic activity of nanozymes, because, sometimes, they showed lower performance and specificity in sensing and biomedicine. Yu et al. [126] covered this issue by studying the impact of citrate, dextran, polylysine and glycine coating on peroxidase mimetic IONPs. In this work, they were able to detect the two-step process of glucose and suggested the idea of using nanozymatic activity extended to other biomedical applications. It is an advanced research area with nanohybrid IONP synthesis. A lot work has yet to be achieved in nanohybrids (conjugated NPs) and the foundation of novel nanodevice and nanosensor creations.

#### 4.1.2. Hyperthermia to Cure Cancer Using IONPs

Surgery, chemotherapy and radiation are the main forms of cancer treatments today; however, none of these are fully established methods [127,128]. In cancer hyperthermia, tumor cells are selectively burnt using a magnetically induced heating effect. Ferromagnetic materials (like Fe_2_O_3_, Fe_3_O_4_) possess hysteretic properties under a time-varying magnetic field, which gives rise to magnetically induced heating [127,128]. This heat conducts into the immediately surrounding diseased tissue whereby, if the temperature can be maintained above the therapeutic threshold of 42°C for 30 min or more, the cancer is destroyed [127,129]. Experimental investigations of the application of magnetic materials for hyperthermia was carried out as early as 1957 when Gilchrist et al. [127] heated various tissue samples with 20–100 nm size particles of γ-Fe_2_O_3_ with a magnetic field of 1.2 MHz.

Until now, hyperthermia is not accepted in any branch of oncology due to the lack of a real clinical effect. As of today, hyperthermia is used in some hospitals as adjuvant therapy, i.e., in conjunction with radiation therapy or chemotherapy in order to reduce the dosage of such treatments. The hyperthermia route cannot differentiate normal and cancer cells. In the existing state, hyperthermia is difficult to be achieved safely without harming or with very less damage to normal tissues, and this is the one of the main problems that needs to be solved [127,128]. With the achievement of the following conditions, IONP hyperthermia may become a successful means to be used as standalone therapy for cancer treatment: sufficient and high nanoparticle concentration should be at the tumor site rather than the surroundings; nanoparticles with high SAR (specific absorption rate) must be used; and SAR parameters must be maximized to obtain heating (with an increase in the coercivity SAR value of nanoparticles).

#### 4.1.3. Drug Delivery

A targeted approach is completed by specific attachment of IONPs to the desired molecule that is specific to the diseased area. Magnetic IONPs are specifically important for such a purpose—in that they are reported for targeted drug delivery in addition to cancer hyperthermia treatment and MRI [130,131,132,133]. These IONP-based nanocarriers are specific and effective in reducing the toxic effects on normal tissues [134], and they minimize the undesired interactions with other molecules [135]. The interaction bond between the anticancer drug“doxorubicin (DOX)” and IONPs is a covalent bond to create electrostatic interactions with negatively charged groups in magnetic nanocarriers and could be a promising nanosystem for acidic solid tumor treatment [136,137]. In recent studies, IONPs have been used with different shells and anticancer agents such as paclitaxel [138,139], methotrexate [140], gemcitabine [141], mitoxantrone [142], epirubicin [143], carmustine [144], cytarabine [145], 5-fluorouracil [146], docetaxel [147] and β-cyclodextrin [148]. Due to specific chemical conditions (such as high pH), in the part affected (cancer affected part/tumor), the drug is released from the nanoparticle surface and induces the required effect of killing the cancer cells [130,133]. To establish an IONP-mediated, successful targeted drug delivery system, key features will be taken into account: first, size-selective synthesis of IONPs, then, interaction, stability, aggregation affects in in vivo conditions.

#### 4.1.4. Alternative Immunosuppressive Activity of IONPs

When a high concentration of immunosuppressants is given to a patient, it may produce serious secondary complications to the patients of autoimmune diseases and transplantations [149]. To lower such risks, an approach was introduced where a nanosystem composed of silica (SI)-coating IONPs that can carry the mycophenolic acid (MPA) as a carrier to the main component of the immunosuppressive mycophenolate mofetil was formulated [150]. Hydrophobic interactions made the bounding of MPA to the SI-coated IONPs possible. This nanosystem was biocompatible at a concentration of 0.56 mg/L with a capacity to transport up to 30% of the MPA’s weight. This concentration of the IONPs–SI–MPA nanosystem made the reduction of the secretion of human interleukin 2 and tumor necrosis factor-α possible to activate the immune cells.

#### 4.1.5. Anticonvulsant Activity of IONPs

There is another very impressive, novel and non-invasive approach of treatment for pharmacological resistance-associated temporal lobe epilepsy. A nanosystem made by covalently attaching anti-interleukin-(IL)-1β monoclonal antibody (1-β mAb) to IONPs functionalized with PEG was injected into the caudal vein of rats with acute temporal lobe-induced epilepsy [151]. MRI showed a higher number of IONP-anti (IL)-1β mAb-PEG in epileptogenic tissues when compared to IONPs alone and the control group in saline solution and demonstrated a higher neuroprotective effect. The drug-resultant convulsion model was analyzed for its effect in in vivo by SI-coated IONPs loaded with antiepileptic phenytoin (PHT). The nanosystem was capable of carrying 250 µg of PHT per 100 mg of nanoparticles [152]. In comparison to the group of rats receiving pure saline solution, the IONPs–SI–PHT nanosystem significantly increased the “after discharge threshold values”, which indicated its potential to reduce the neural excitability and the incidence of convulsion [152].

#### 4.1.6. Antifungal Activity of IONPs

Fungal diseases are generallya type of infection that affects a patient but can turn lethal when not treated properly. Nystatin (NYS) is one of the most common fungicides. A nanosystem by combining IONPs, NYS and CS (chitosan) was prepared by Hussein-Al-Ali et al. [153]. They demonstrated that the release profile of the IONPs–CS–NYS nanosystem is 1800 min, while NYS in physical mixture can only last about 20 min, which is comparatively lower than the IONPs–CS–NYS nanosystem. The reason behind this controlled release of antifungal activity is explained by the electrostatic interaction between positive charge of CS and negative charge of NYS. Another example of antifungal drugs is ketoconazole and amphotericin B; they have been tested by a specific magnetic nanosystem to improve their antifungal activities with decreased side effects. Epoxy-functionalized IONPs immobilized with HSA coupled to ketoconazole by hydrophobic interaction for binding [154], and IONPs directly bounded to amphotericin B by reaction first with amide and then with aldehyde groups have been observed [155].

#### 4.1.7. Antibiotic Activity of IONPs

Highly resistant bacterial strains and their limited conventional antibiotics necessitated the development of a design of antibiotic-carrier nanosystems. Specifically designed antibacterial nano agents proved to be an advantage because of their qualities, i.e., large surface area, biocompatibility, response to magnetic fields and recyclability.In a report, IONPs have been functionalized with CS to be used as carriers of streptomycin [153,156]. In a normal physical mixture with phosphate-buffered saline, the streptomycin-release effect lasted for 20 min, while its complete release lasted for 350 min when exposed as a nanosystem. This indicates the ability of IONPs to act as a controlled-release system [150]. Studies have been conducted with different antibiotics such as rifampicin, tetracycline, anthracycline, cephalosporin and fluroquinolone, and they were combined by physical adsorption to silver nanoparticle (Ag)-loaded IONPs [157]. Similarly, doxycycline, ceftriaxone and cefotaxime were also combined to IONP nanosystems attached with silver (Ag) [158]. Many other examples of composed nanosystems have been investigated, for example, amoxicillin, streptomycin, amikacin, vancomycin, bacitracin, cefotaxime, gentamicin, kanamycin, polymyxin, kanamycin and neomycin directly combined with IONPs without using any shell coating [159]. Promising results have been reported in the field of antibacterial activity by these IONPs in vitro, and the expectations remain high for in vivo applications. Since these magnetic IONPs have the capability to expand the understanding of physiochemical and biological properties, they may be of high interest among researchers in the near future [160].

#### 4.1.8. IONPs for Imaging

Magnetic resonance imaging is a great technique to observe the differentiation between pathogenic and healthy tissues. MRI is the most valuable noninvasive technique for imaging due to its tomographic capabilities and high spatial resolution. Recently, IONPs have been of interest as a contrast agent for MRI [161]. Magnetite and maghemite are commonly used IONPs due to their biocompatibility with low toxicity, and under in vivo conditions, they are decomposable. Superparamagnetic iron oxide nanoparticles (IONPs) are very important as MRI contrast agents. The IONPs label has been approved for clinical use. Being highly sensitive to MR detection and because it is non-toxic, it can be used in MRI as a blood pool agent [162]. Recently, developments in MRI imaging have achieved near-microscopic resolution invivo [163].

The introduction of the nanoprobe in MRI is a topic of research gaining particular interest in molecular imaging. These nanoparticles are coupled with antibodies or a ligand inserted in the human body, where they are bound with specific receptors on tumor cells to allow the IONPs to aggregate in tumor tissues. In this condition, IONPs generate magnetism because of the magnetic field to detect and diagnose small lesions with MRI in the early stage.

#### 4.1.9. Cellular Labeling/Cell Separation Using IONPs

**Figure 12:**
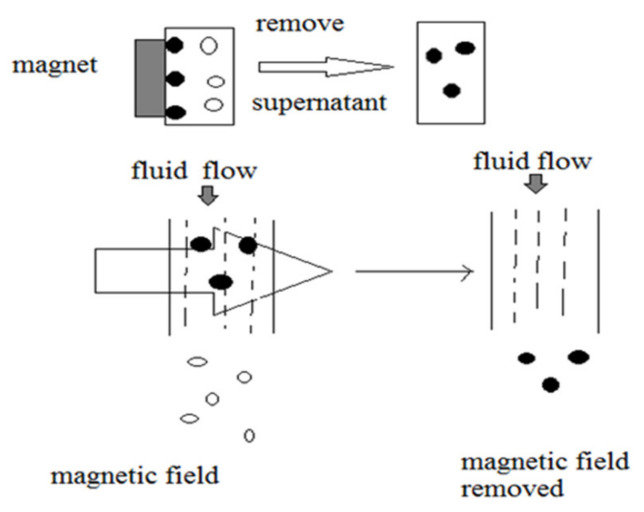
Magnetic separation process. (I) Tagging of desired biological entity with IONPs (•); (II) Separation of desired entity-tagged nanoparticles by fluid-based magnetic process (◦).

Cell separation is the process of removing one cell population from another in vivo. It is a key part of stem cell and oncology research androutine clinical diagnosis. Cell labeling by using ferro/paramagnetic substances is commonly used in vivo for cell separation [164], and MRI detects these labeled cell [165]. Recently, two approaches have been used in these labeling techniques, namely, (a) by attaching magnetic particles to the cell, and (b) by internalizing biocompatible magnetic particles by using fluid phase endocytosis [166] or receptor-mediated endocytosis [167]/phagocytosis [167]. In receptor-mediated endocytosis, the surface of the nanoparticle was modified with a ligand that is compatible with the targeted cells [168]. By using a receptor-mediated approach, the nanoparticles are been labeled by different ligands to attract other potential receptors, i.e., monoclonal antibodies (Mabs) [168]. Lactoferrin, insulin, transferrin, growth factors and albumin on the surface of the mammalian cell are examples of preferential targeting agents [169]. These cellular markers are efficient internalizing molecules bonded with receptors [170]. In various biological processes, separation of different yet specific biological entities from their intrinsic environmental niche is regularly practiced in order to prepare the concentration of the desired cell or biomolecules for further analysis. Magnetic separation of desired biological entities is achieved by making use of biocompatible surface-modified iron oxide nanoparticles (Figure 12).

Assisted reproduction in animals for the improvement of reproduction performance, specific targeting and removal of dead and poor performing spermatozoa from semen samples is carried out by magnetic iron oxide nanoparticles (Figure 7) [171,172,173]. Additionally, IONPs with or without surface coating are used for the separation of biomolecules. Nascent IONPs also have great potential for separation of bacterial DNA and bacteria. Furthermore, the same nanoparticles coated with starch molecules are capable of amylase enzyme isolation from a complex matrix [174,175].

#### 4.1.10. IONP Tissue Engineering/Tissue Repair

The whole idea of tissue repair came from coating the nanoparticles onto the surface of two sites of the tissue at the desired location. With this technique, tissue damage was expected to be minimized by using wavelengths. Lobel et al. [176] repaired tissue with the help of IONPs by placing protein or synthetic polymer-coated nanoparticles between the two tissue surfaces to induce joining of the tissue at temperature greater than 500 °C (temperature known to induce union of the tissue). This whole approach is briefly described as using “magnetized” cells as building blocks to replace damaged tissues. The nanoparticles which can strongly absorb light can also be useful in tissue repairing, for example, gold/silica-coated IONPs [177]. Apart from tissue replacement, it is important for a tissue to mimic the formation, cellular organization and physiology of healthy native cells and their development. Thus, proper control over the cellular process under stem cell differentiation is a major challenge for tissue engineers.

#### 4.1.11. Stem Cell Tracking by IONPs

Stem cell research is an interesting topic for researchers working in the biomedicine field for its differentiation potential and proliferative capacity. In the field of regenerative medicine, MRI helps to check the fate of stem cells after introduction to the body. It is necessary to have cell labeling which is nontoxic, stable and effective to achieve good results in monitoring migration, differentiation and treatment mechanisms of stem cells. Two methods have been popular in the field of stem cell tracking, i.e., intracellular labeling and cell surface labeling. Tracking stem cells can be achieved easily by developing a non-immunogenic system which is compatible with the cell pathways. By using mice, the tracking of transplanted cells and study of a differentiation process has become possible. Anderson et al. [178] investigated bone marrow cells by SPIO-labeled Sca1+ into glioma-bearing severe combined immunodeficient (SCID) mice. During the tumor growth, MRI was performed. Mice with labeled cells showed hypointense regions within the tumor which evolved to develop a dark hypointense ring. In addition, histological analysis showed that the transplanted cells detected in the tumor were differentiated to endothelial-like cells. This tumor vasculature contributed to ongoing revascularization and angiogenesis [179]. It is still important to develop a gene-targeted probe for the multigenicity of tumor cells, and this remains an ongoing challenge.

#### 4.1.12. Transplant Monitoring by IONPs

Iron oxide nanoparticles have been used for in vivo studies to monitor tissue transplantation, e.g., pancreatic islets in diabetes therapy. Pinaud et al. [180] used dextran coating on cy5.5 (cyanine 5.5)-modified iron oxide nanoparticles. They were implanted under kidney capsules, MRI examination was performed for six months. Implantation under kidney capsules and intraportal infusion into diabetic mice models showed restoration of normoglycemia. The ability of the transplanted islets to secrete insulin was confirmed by performing ex vivo microscopic studies.

A complete study on the monitoring of pancreatic islets was performed by Wu et al. [181]. The commercial iron oxide nanoparticles (Feridex formulation) were compared with non-hydrolytically (thermal decomposition in the presence of PVP) synthesized iron oxide nanoparticles with a diameter of 5–8 nm. Higher iron accumulation was proven for PVP–SPIO nanoparticles in the commercial Feridex formulation. According to this study, a possibility has been revealed to detect very low levels of transplanted islets by MRI analysis in vivo.

#### 4.1.13. Fluorescence Techniquesand Encapsulated IONPs

Luminescent IONPs give good reaction homogeneity and fast reaction kinetics because of their small size and large surface area-to-volume ratio than other microbeads. However, while there are limited applications by cellular imaging techniques in bioanalysis, the external magnetic field can be manipulated without using centrifuge and filtration techniques by using magnetic luminescent nanoparticles [182]. Optical characteristics, i.e., sharp emission, long lifetime and photo stability provide the implementation of an internal calibration in a detection system. This allows a unique way to control quality and much easier quantification in multiplexed immune analysis processes. By using these methods, it is possible to detect biological threats by simple quantitative multiplex protein analysis using conventional organic dyes and application in disease diagnosis. Polystyrene magnetic beads are an example of magnetic fluorescent particles which are entrapped in organic dyes (like quantum dots) or shells of quantum dots [183]. Another example of fluorescence application is dye-doped silica shell coating on iron oxide particles, and iron oxide and Quantum dots-embedded nanoparticles are easier [184].

Encapsulation of IONPs is another modification and stabilizing strategy. IONP encapsulation by inorganic polymers such as silica and metals and core/shell nanostructures allows for the tuning of magnetic properties, stability and functionalization for their specific applications in separation and detection processes. However, biocompatible polymers or inorganic compounds are the best choice. Polymeric matrix encapsulation increases compatibility with organic ingredients; protects the surface of the particle from oxidation; and reduces leaching susceptibility, toxicity and chemical stability [185]. Biocompatible hydrophilic shell encapsulation gives promising results in terms of modification of IONPs. There are many examples of typical shell materials, such as water soluble polymer matrixes, amphiphilic ligands and hydrophilic inorganic materials. Encapsulation improves chemical stability, reduces toxicity and improves the ability to disperse [186,187]. Cross linking of polymers is a very good method used for the encapsulation of IONPs. These include encapsulation by chitosan [188]; co-polymers such as poly (maleis anhydridealt-1-octadecene)-PEG [189], polystyrene-*co*-PEG (PS-*co*-PEG) [190], polysaccharides [191], polyaspartate [192]; and inorganic materials like gold [193].

## 5. Future Directions

Major clinically available applications of IONPs are nucleic acid detection and detection of infectious bacteria and viruses. Rapid and ultrasensitive detection of disease causing agents (bacteria/viruses) is intensely important at POC (point of care). IONPs can play a key role in these perspectives.

Considering the recent ongoing example of the COVID-19 pandemic that has shown the need to develop detection techniques, it is also important that detection technology should be user friendly, low cost and possess easy availability. Nanoparticle-based detection approaches are found to be ideal in the case of rapidity [194]. Some of the promising ideas can be seen by incorporating IONPs in tools such as RT-PCR [195] SERS (surface-enhanced Raman spectroscopy) [196,197,198] etc. Recent studies show that a magnetic IONP-based SERS probe (Figure S8 as represented in additional materials) would be an encouraging approach in POC, i.e., point of care diagnostics [199]. Both Fe_2_O_3_ and Fe_3_O_4_ nanoparticles, also combining with Au (gold), could give a combined effect of magnetic properties and optical properties [200]. This and other possible combinations can offer tremendous possibilities not only for SERS but also in cancer hyperthermia, MRI, photothermal therapies, etc.

Zhao et al. [195] in his recent study used magnetic IONPs for RNA extraction of COVID-19.RT PCR could also be developed with IONPs–RNA mixtures by which time in amplification can be reduced. IONP-based RNA extraction can be a great idea that can be followed by RT PCR or SERS studies. As IONPs in the colloidal form can make an SERS substrate, specific attachment of RNA to IONPs could be detected by RT PCR as well as SERS, as shown in Figure S8 (additional materials).

IONP-based MOFs (metal–organic frameworks) maybe promising candidates in biomedical applications, as they can be easily implanted in the body and removed when their purpose is achieved. From the detection perspective, their interconnected porosity allows them to target (pathogens/viruses/biomolecules) in an optimum and large manner. The target can also be adsorbed or absorbed on the surface of MOFs, and adding to this high surface-to-volume ratio will make them potential candidates in applications containing adsorption phenomenon [194]. In addition to the above innovative applications, there is a need to develop commercial technologies based on IONPs relating to various fields such as biomedicine, electronics, catalysis etc.

## 6. Conclusions

The present article briefly presents different synthesis methods for IONPs. Different strategies, factors and characterizations to confirm phase purity in the IONP synthesis field are also reviewed. Significant applications of IONPs are also discussed, therefore contributing to research regarding IONP synthesis.

Synthesis and applications of IONPs have been of interest in the field of nanotechnology over the past decade. Significant work has been reported on the synthesis and applications of IONPs. Different synthetic routes have been adapted, but a major challenge in the synthesis field that remains is the obtention of size- and phase-controlled synthesis with reproducibility. Reproducibility is difficult to achieve in the passive approach of existing synthesis methods in which the partial mixing of a reactant takes place and undesired reactions take place, affecting the properties of the nanoparticles. To overcome the existing challenges in controlled reproducible synthesis, ideally, the following points should be fulfilled: (a) direct active and complete mixing of reactants; (b) automation and (c) enabled reaction parameters controlled precisely. Each synthesis method has its own pros and cons. In the case of characterization, it is very difficult to identify oxides phases of iron with the help of a single characterization tool; however, this problem can be solved by using multiple characterization techniques to identify and confirm the oxide phase of iron. Among iron oxides; γ-Fe_2_O_3_ and Fe_3_O_4_ are widely used materials. In the IONP synthesis area, a clear reaction mechanism is also important as it enables in-depth understanding of the reaction so as to gain good control over the synthesis parameters and hence control of the nanoparticles’ size and properties.

We have covered the areas of systematic preparation of IONPs and their administration for use in various applications such as biosensing, solar energy storage devices, hyperthermia, MRI, drug delivery, alternative immunosuppressive applications, anticonvulsant applications, antifungal applications, antibiotic applications, cell labeling/cell separation, tissue repair, cell tracking, transplant monitoring, environmental remediations etc. Utilizing magnetic nanoparticles proved to be a promising approach for in vivo as well as in in vitro studies. Magnetic nanoparticles showed great results in the field of medicinal and healthcare treatment due to their low toxicity, biocompatibility and ability to be manipulated by the application of a magnetic field. However, they showed several limitations, especially when in the case of in vivo studies. The application of IONPs, in vivo approaches still requires more effort to achieve the hope for tomorrow, especially in the area of life science approaches when compared to in vitro studies. Preclinical studies tend to show great interest in cancer, hyperthermia, drug delivery, tissue repair mechanisms, and stem cell technologies with the objective of achieving clinical feasibility. In this review, we underline the limitations of the use of IONPs and highlight the potential improvements offered by IONPs in various areas.

## Figures and Tables

**Table 1:** Merits and demerits of different IONP nanoparticle synthesis methods.

Type of Synthesis	Pros	Cons	Reference
Microwave	Short reaction time, higher yields, excellent reproducibility, easy handling	Expensive, unsuitable for scale-up and reaction monitoring	[28,29]
Spray pyrolysis	Finely dispersed particles of predictable size, shape and variable composition	Aggregated particles, expensive	[30,31,32]
Laser pyrolysis	Small particle size, narrow particle size distribution, near absence of aggregation	Complicated, very expensive	[21,31]
Pulsed wire discharge method	Fast process, higher purity of NPs	Batch process, limited production, high vacuum systems, costly process, contaminations in product	[33,34]
Chemical vapor condensation	Suitable for preparing small quantities to demonstrate desired properties in the laboratory	Low production, difficult to control size and particle size distribution	[35]
Co-precipitation	Convenient method, simple and rapid preparative method, easy control of particle size and composition	Extensive agglomeration, poor morphology and particle size distribution	[36,37,38]
Thermal decomposition	Producing highly monodispersed particles with a narrow size distribution	High cost, long-time synthesis reaction, high temperature	[39,40,41]
Microemulsion	Monodispersed nanoparticles with various morphology can be produced	Not very efficient and difficult to scale up	[39,42]
Polyol	Uniform size particles can be prepared, easy to scaleup	Needs high temperature, long time	[22,31]
Sol–Gel	Low processing cost, energy efficiency, high production rate, and rapid productivity	Limited efficiency, high cost	[43,44,45]
Sonochemical	Simple, low cost, safe, environment friendly, absence of many reactants	Very small concentration of prepared NPs, particle agglomeration is very narrow	[33,46]
Biological synthesis of nanoparticles using plants and bacteria	Selectivity and precision for nanoparticle formation, cost effective, eco friendly	Limited knowledge, difficulty in controlling size and properties	[47,48]

**Table 2:** Limitations of co-precipitation and the polyol method.

Co-Precipitation Method	Polyol Method
⮚ pH control is necessary, but it is difficult ⮚ Fe_3_O_4_ nanoparticles with stoichiometric composition are difficult to synthesize by this method	⮚ Mechanism leading to Fe_3_O_4_ formation is not clear yet in this method ⮚ Origin of oxygen element in magnetite is not clear

**Table 3:** Reagents/precursors used in various IONP synthesis methods.

Reagents/Precursors	Used Concentration	Synthesized Nanoparticles	Reference
Precursor A: Fe(III)(NO_3_)_3_·9H_2_O Precursor B: Fe(II)–naphthenate	0.65 M 0.9 M	FeO	[52]
Goethite FeO(OH) Oleic acid	3 mM 13.5 mM	FeO	[53]
Fe(stearate)_2_ i.e., ferric stearate oleic acid	2.22 mM 4.44 mmol	FeO	[55]
Fe(III) acetylacetonate Oleic acid Oleylamine	0.75 mM 5 mM 5 mM	core/shell FeO/Fe_3_O_4_	[51]
Fe(NO_3_)_3_· 9H_2_O C_6_H_8_O_7_·H_2_O	0.1M 0.05 to 0.2M	Fe_2_O_3_ (admixture of α and ɤ)	[57]
Precursor solution of Fe (III) NaOH	1 M 6 M	α-Fe_2_O_3_	[58]
Poly vinyl alcohol Fe(NO_3_)_3_ EDTA	0.25 M 0.1 M 0.1 M	α-Fe_2_O_3_	[59]
Fe_2_(SO_4_)_3_ and FeSO_4_	2:1 Fe(III) to Fe(II)		
	Total Fe of 8.6 × 10^−3^M	ɤ-Fe_2_O_3_	[62]
NH_4_OH	25% aqueous solution		
FeCl_3_·6H_2_O FeCl_2_·4H_2_O KCl KOH	1.35 g 0.50 g 3.9 g 1.22 g	IONPs	[64]
FeCl_2_·4H_2_O and FeCl_3_·6H_2_O	Fe^2+^/Fe^3+^=1/2		
	dissolved in 2 M HCl	ɤ-Fe_2_O_3_	[66]
NaOH	2 M		
FeCl_2_·4H_2_O and FeCl_3_·6H_2_O NH_4_OH HNO_3_ HCl	Molar ratio 2:1 28% 65% 37%	ɤ-Fe_2_O_3_	[67]
FeCl_3_·6H_2_O FeCl_2_·4H_2_O NH_4_OH	0.1 M 0.1 M 10% solution	ɤ-Fe_2_O_3_	[68]
FeCl_3_∙6H_2_O FeCl_2_∙4H_2_O HCl N(CH_3_)_4_OH.	1 M 2 M 2 M 25 % aqueous	Fe_3_O_4_	[78]
FeCl_2_∙4H_2_O FeCl_3_∙6H_2_O NaOH	0.01 M 0.02 M 0.08 M	Fe_3_O_4_	[79]
Fe(acac)_3_ C_6_H8O_6_ Ultrapure water	30 mM/15 mM/50 mM 0.025 M/0.005 M/0.05 M 12 M/1.2 M	Fe_3_O_4_	[22,25,26,27]

**Table 4:** Different areas of application of magnetic nanoparticles.

Discipline	Application	Reference
Environmental Remediation	Waste water treatment: adsorption, membrane filtration, permeable reactive barriers, photocatalysis, dematerialization (reduction in material quantity). Sensing: nonporous membranes, pollutant sensors, nanowire sensor for explosives. Energy: heat distribution, e.g., ceramic-like materials that provide sufficient durability and reliability to the entire structure. Pollution prevention: remediation, detection and monitoring.	[95,100,101,102,103]
Biomedical	Magnetic hyperthermia, controlled drug release, magnetic separation, MRI contrast agent, controlled drug release, cell separation and handling of cells, cell labeling, tissue repair, antiviral.	[104,105,106,107]
Defense and Aerospace	Fuel additives, energy devices, nanocomposites, sensors, nanocoatings and electronics.	[108,109,110]
Construction	Coloring concrete, tiles, bricks and other construction materials. Nanoscale sensors, nanocoatings, smart materials, nanocomposites.	[105,111]
Electronics	Nanoscale memory, spintronics, printed electronics, nanowires and NEMS (nano electro mechanical systems)	[112,113]
Healthcare	Nanoscale biosensors and imaging, antimicrobial activities; nanophotothermolysis with pulsed lasers, antiviral, preventing skin aging; implants; nanocarrier for vaccination	[114,115]
Automotive Textiles	Additive in catalysts and lubricants, sensors, nanofibers, coatings, composite fillers, fuel cells and smart materials	[116]
Agriculture and Food	Nanopesticides, nanofungicides, nanofertilizers, nanosensors, nanofood, encapsulation, gene transfer (crop improvements), food packing	[117,118,119,120]

## Supplementary Materials

The following are available online at https://www.mdpi.com/1996-1944/13/20/4644/s1. Figure S1: Microwave method for nanoparticle synthesis; Microwaves radiations with frequency ranges from 300 MHz to 300 GHz are used in reaction solution (Most commonly 2.456 GHz is used), Figure S2: Spray pyrolysis method for nanoparticle synthesis; Aerosol droplets undergo evaporation of solvent followed by solute condensation and drying followed by thermolysis of the precipitated particles at high temperature, Figure S3: Co-precipitation method for nanoparticle synthesis; Co-precipitation of various salts like nitrates, sulphates, chlorides, perchlorates etc. is carried out. This co-precipitation is carried out under fine control of pH by using solutions of NaOH, NH4OH to yield corresponding oxide nanoparticles, Figure S4: Microemulsion method for nanoparticle synthesis; When micro emulsion containing reactants are mixed together, due to the reaction micro droplets are formed. Due to the presence of surfactant, fine micro droplets of aqueous get trapped within surfactant molecule assembles. Micro cavities stabilized by surfactant can provide locking up effect that limits particle nucleation, growth, agglomeration results in nanoparticle formation, Figure S5: Polyol method for nanoparticle synthesis; Polyols also control particle growth and prevent aggregation of particles. Precursor is suspended into liquid polyol and system is heated up to boiling point of polyol. Precursor is reduced to nuclei and subsequently nanoparticles synthesized, Figure S6: Sol gel method for nanoparticle synthesis; Mainly three steps: (1) Preparation of sol, (2) Successive gelation, (3) Removal of solvent, Various nanocomposites either crystalline or amorphous can be synthesized with controlled porosity in bulk amounts by using this method, Figure S7: Thermal decomposition method for nanoparticle synthesis; Thermal decomposition of a metallic precursor in presence of organic surfactant is carried out. Synthesis is generally carried out at high temperature. Highly monodispersed particles with a narrow size distribution can be synthesized with this method, Figure S8: IONPs based SERS i.e., Surface Enhanced Raman Spectroscopy (a), IONPs based nucleic acid extraction (b).
