# Supplementary material for: Magnetic Iron Oxide Nanoparticle (IONP) Synthesis to Applications: Present and Future
Source: Materials (Basel). 2020 Oct 18;13(20):4644. doi: 10.3390/ma13204644 (PMC7603130; doi:10.3390/ma13204644)
Supplement: Supplementary file 1 [file materials-13-04644-s001.pdf]

materials

Supplementary

Magnetic Iron Oxide Nanoparticle (IONP)Synthesis to Applications: Present and Future

Nene Ajinkya [1,*], Yu Xuefeng [1], Poonam Kaithal [2], Luo Hongrong [1], Prakash Somani [3] and Seeram Ramakrishna [4]

[1] Materials and Interfaces Center, Shenzhen Institutes of Advanced Technology, Chinese Academy of Sciences, Shenzhen 518055, China; xf.yu@siat.ac.cn (Y.X.); hluo@scu.edu.cn (L.H.)
[2] Department of Molecular and Cellular Engineering, Dear Dr. Czerwinski, Jacob Institute of Biotechnology and Bioengineering, SHUATS, Allahabad 211007, India; poonam.kaithal@gmail.com
[3] Center for Grand Challenges and Green Technologies, Applied Science Innovations Pvt. Ltd., Pune 411041, India; spsomani97@gmail.com
[4] Center for Nanofibers and Nanotechnology, National University of Singapore, Singapore 117576; seeram@nus.edu.sg
* Correspondence: neneajinkya@gmail.com

Received: 13 August 2020; Accepted: 20 September 2020; Published: date

Nanoparticle synthesis methods

Microwave method[1]:

- ✓ Using microwave radiations in reaction solution to synthesize nanoparticles
- ✓ A simple rapid and easy
- ✓ Fast and homogenous conditions can be maintained

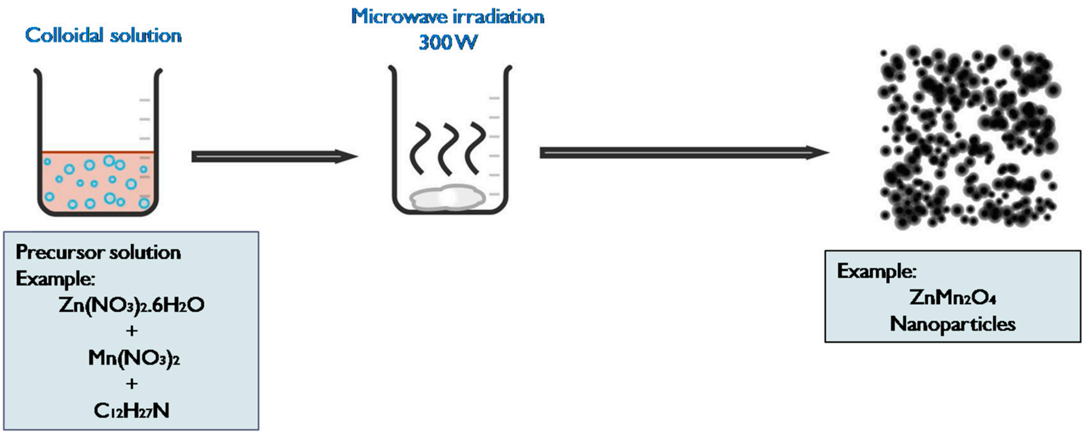

Figure S1. Microwave method for nanoparticle synthesis; Microwaves radiations with frequency ranges from 300 MHz to 300 GHz are used in reaction solution (Most commonly 2.456 GHz is used).

Spray pyrolysis[2]:

- ✓ Can control parameters; homogenous well- defined nanoparticles
- ✓ High production rate
- ✓ Rapidity and continuity

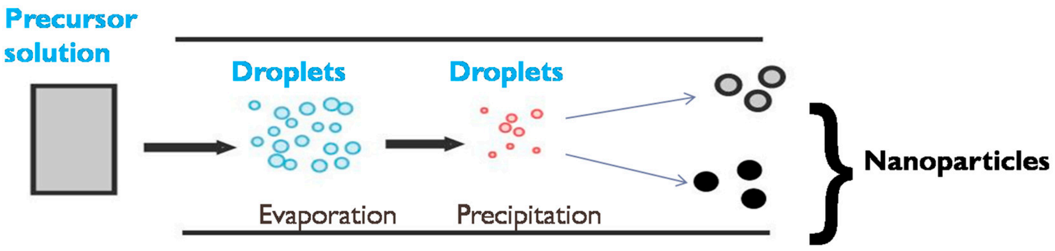

Figure S2. Spray pyrolysis method for nanoparticle synthesis; Aerosol droplets undergo evaporation of solvent followed by solute condensation and drying followed by thermolysis of the precipitated particles at high temperature.

Co-precipitation[3]:

- ✓ A simplest method
- ✓ pH control is main factor
- ✓ Reaction needs low temperatures

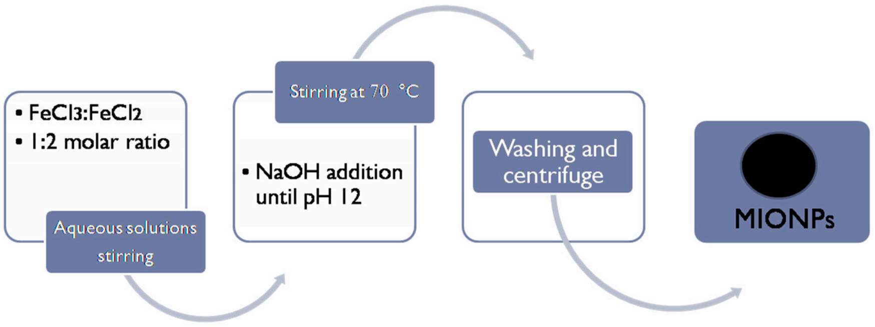

Figure S3. Co-precipitation method for nanoparticle synthesis; Co-precipitation of various salts like nitrates, sulphates, chlorides, perchlorates etc. is carried out. This co-precipitation is carried out under fine control of pH by using solutions of NaOH, NH_4OH to yield corresponding oxide nanoparticles.

Microemulsion method[4]:

✓ Monodispersed Nanoparticles with various morphology can be produced

✓ difficult to scale up

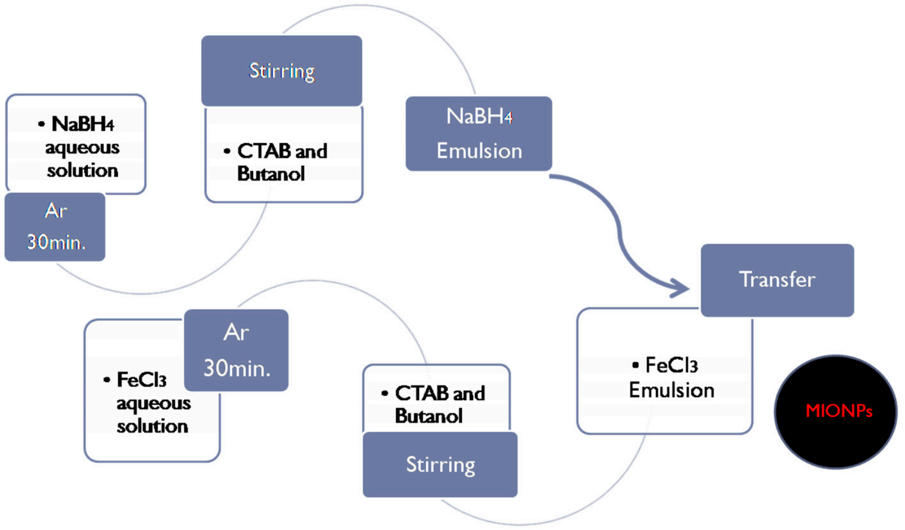

Figure S4. Microemulsion method for nanoparticle synthesis; When micro emulsion containing reactants are mixed together, due to the reaction micro droplets are formed. Due to the presence of surfactant, fine micro droplets of aqueous get trapped within surfactant molecule assembles. Micro cavities stabilized by surfactant can provide locking up effect that limits particle nucleation, growth, agglomeration results in nanoparticle formation.

Polyol method[5]:

✓ Polyols act as reducing agent and high boiling solvent

✓ A wide variety of inorganic non aggregated particles can be prepared

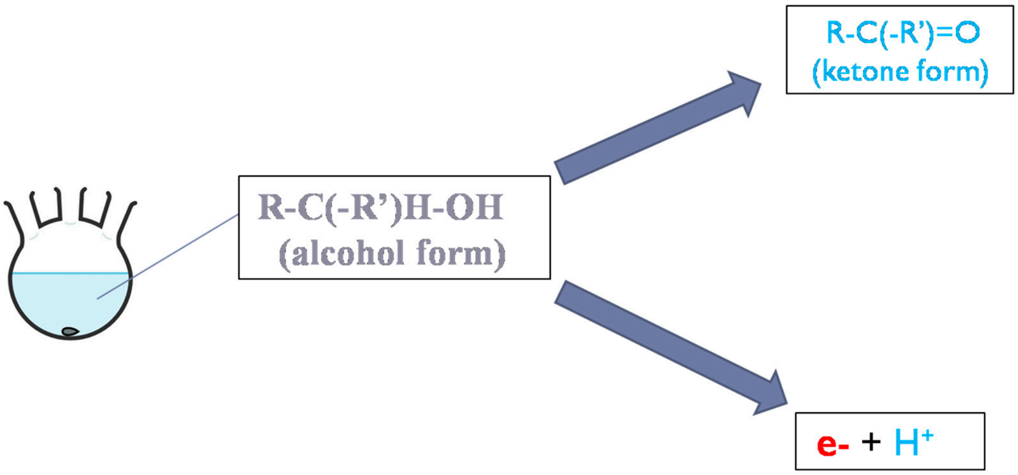

Figure S5. Polyol method for nanoparticle synthesis; Polyols also control particle growth and prevent aggregation of particles. Precursor is suspended into liquid polyol and system is heated up to boiling point of polyol. Precursor is reduced to nuclei and subsequently nanoparticles synthesized.

Sol gel method[6]:

- ✓ High production rate, and rapid productivity of fine homogeneous particles
- ✓ Low processing cost
- ✓ Effective method and widely used in synthesis of metal oxides

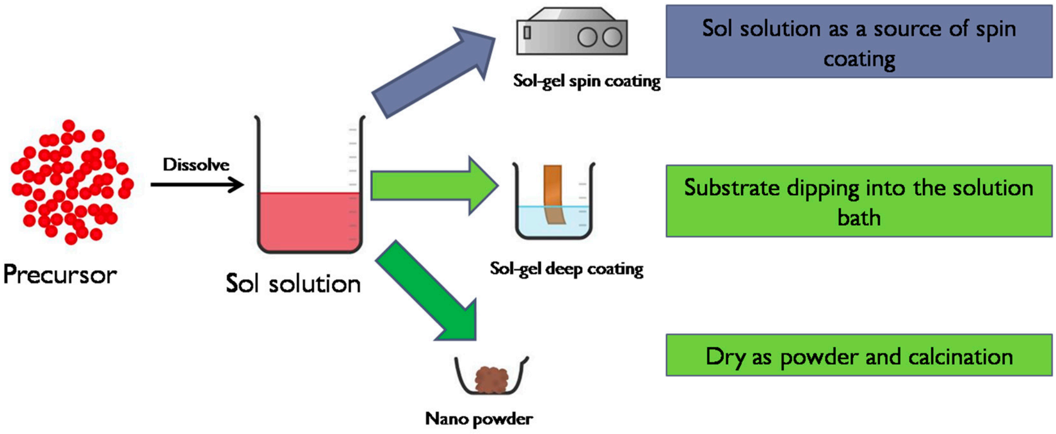

Figure S6. Sol gel method for nanoparticle synthesis; Mainly three steps: (1) Preparation of sol, (2) Successive gelation, (3) Removal of solvent, Various nanocomposites either crystalline or amorphous can be synthesized with controlled porosity in bulk amounts by using this method.

Thermal decomposition[7]:

Can synthesize
- ✓ Highly monodispersed particles
- ✓ With a narrow size distribution

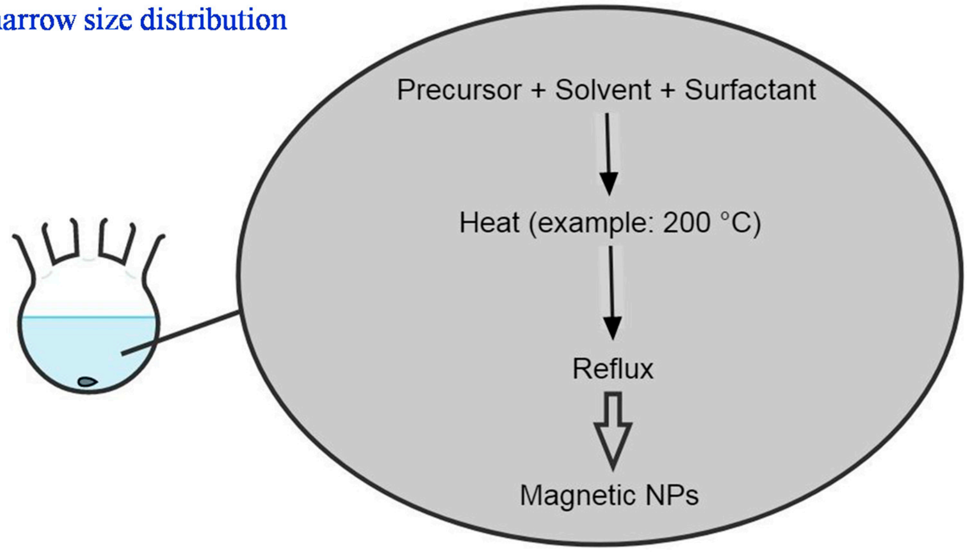

Figure S7. Thermal decomposition method for nanoparticle synthesis; Thermal decomposition of a metallic precursor in presence of organic surfactant is carried out. Synthesis is generally carried out at high temperature. Highly monodispersed particles with a narrow size distribution can be synthesized with this method.

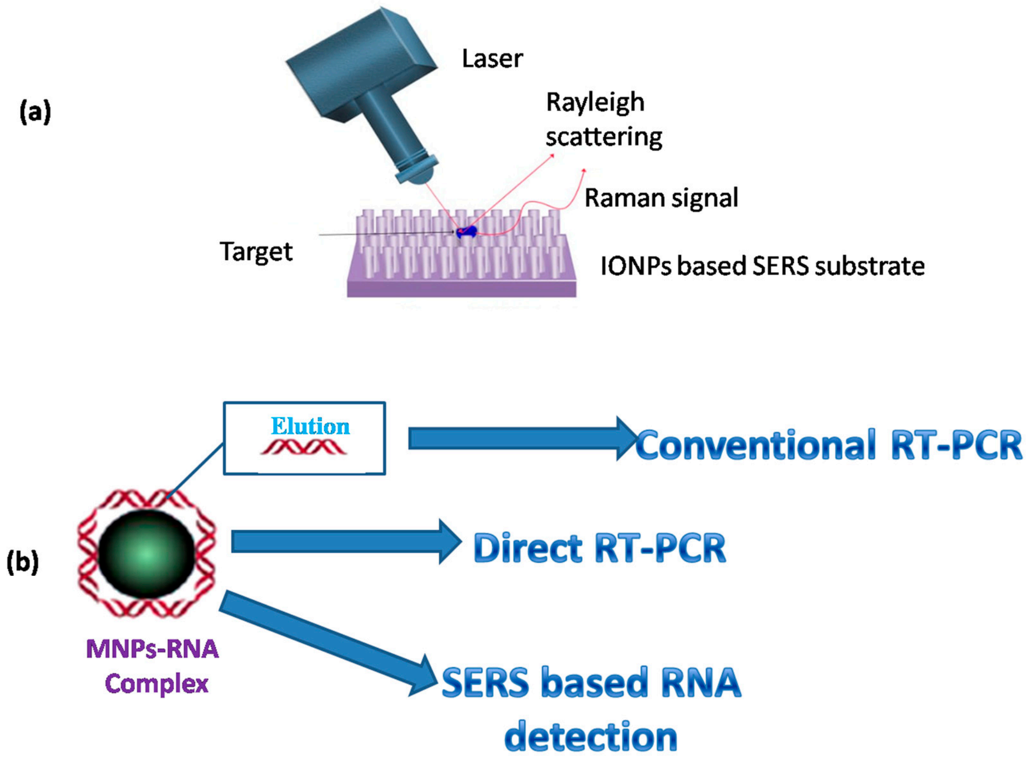

Figure S8. IONPs based SERS i.e. Surface Enhanced Raman Spectroscopy (**a**), IONPs based nucleic acid extraction (**b**).

References

1. Morán-Lázaro, J.P.; Guillen-López, E.S.; López-Urias, F.; Muñoz-Sandoval, E.; Blanco-Alonso, O.; Guillén-Bonilla, H.; Guillén-Bonilla, A.; Rodríguez-Betancourtt, V.M.; Sanchez-Tizapa, M.; Olvera-Amador, M.D.L.L. Synthesis of znmn2 o4 nanoparticles by a microwave-assisted colloidal method and their evaluation as a gas sensor of propane and carbon monoxide. *Sensors (Switzerland)* **2018**, *18*, doi:10.3390/s18030701.
2. Eslamian, M.; Ahmed, M.; Ashgriz, N. Modelling of nanoparticle formation during spray pyrolysis. *Nanotechnology* **2006**, *17*, 1674–1685, doi:10.1088/0957-4484/17/6/023.
3. Hariani, P.L.; Faizal, M.; Ridwan, R.; Marsi, M.; Setiabudidaya, D. Synthesis and Properties of Fe_3O_4 Nanoparticles by Co-precipitation Method to Removal Procion Dye. *Int. J. Environ. Sci. Dev.* **2013**, *4*, 336–340, doi:10.7763/ijesd.2013.v4.366.
4. Ansari, S.A.M.K.; Ficiarà, E.; Ruffinatti, F.A.; Stura, I.; Argenziano, M.; Abollino, O.; Cavalli, R.; Guiot, C.; D'Agata, F. Magnetic iron oxide nanoparticles: Synthesis, characterization and functionalization for biomedical applications in the Central Nervous System. *Materials* **2019**, *12*, 465, doi:10.3390/ma12030465.
5. Nene, A.G.; Takahashi, M.; Wakita, K.; Umeno, M. Size controlled synthesis of Fe_3O_4 nanoparticles by ascorbic acid mediated reduction of Fe(acac)3 without using capping agent. *J. Nano Res.* **2016**, *40*, 8–19, doi:10.4028/www.scientific.net/JNanoR.40.8.
6. Thiagarajan, S.; Sanmugam, A.; Vikraman, D. Facile Methodology of Sol-Gel Synthesis for Metal Oxide Nanostructures. *IntechOpen* **2017**, 38, doi:10.5772/intechopen.68708.
7. Campanini, M.; Ciprian, R.; Bedogni, E.; Mega, A.; Chiesi, V.; Casoli, F.; De Julián Fernández, C.; Rotunno, E.; Rossi, F.; Secchi, A.; et al. Lorentz microscopy sheds light on the role of dipolar interactions in magnetic hyperthermia. *Nanoscale* **2015**, *7*, 7717–7725, doi:10.1039/c5nr00273g.

© 2020 by the authors. Submitted for possible open access publication under the terms and conditions of the Creative Commons Attribution (CC BY) license (http://creativecommons.org/licenses/by/4.0/).
